# Machine learning-based prognostic modeling of lysosome-related genes for predicting prognosis and immune status of patients with hepatocellular carcinoma

**DOI:** 10.3389/fimmu.2023.1169256

**Published:** 2023-05-19

**Authors:** Wenhua Li, Qianwen Wang, Junxia Lu, Bin Zhao, Yuqing Geng, Xiangwei Wu, Xueling Chen

**Affiliations:** ^1^ Key Laboratory for Prevention and Treatment of High Morbidity in Central Asia, National Health and Health Commission, Shihezi, China; ^2^ Department of Immunology, Shihezi University School of Medicine, Shihezi, China; ^3^ The First Affiliated Hospital, Shihezi University School of Medicine, Shihezi, China

**Keywords:** hepatocellular carcinoma, lysosome, machine learning, prognostic model, RAMP3, immune infiltration, drug sensitivity

## Abstract

**Background:**

Hepatocellular carcinoma (HCC) is a leading cause of cancer-related deaths worldwide. Lysosomes are organelles that play an important role in cancer progression by breaking down biomolecules. However, the molecular mechanisms of lysosome-related genes in HCC are not fully understood.

**Methods:**

We downloaded HCC datasets from TCGA and GEO as well as lysosome-related gene sets from AIMGO. After univariate Cox screening of the set of lysosome-associated genes differentially expressed in HCC and normal tissues, risk models were built by machine learning. Model effects were assessed using the concordance index (C-index), Kaplan-Meier (K-M) and receiver operating characteristic curves (ROC). Additionally, we explored the biological function and immune microenvironment between the high- and low-risk groups, and analyzed the response of the high- and low-risk groups to immunotherapy responsiveness and chemotherapeutic agents. Finally, we explored the function of a key gene (RAMP3) at the cellular level.

**Results:**

Univariate Cox yielded 46 differentially and prognostically significant lysosome-related genes, and risk models were constructed using eight genes (RAMP3, GPLD1, FABP5, CD68, CSPG4, SORT1, CSPG5, CSF3R) derived from machine learning. The risk model was a better predictor of clinical outcomes, with the higher risk group having worse clinical outcomes. There were significant differences in biological function, immune microenvironment, and responsiveness to immunotherapy and drug sensitivity between the high and low-risk groups. Finally, we found that RAMP3 inhibited the proliferation, migration, and invasion of HCC cells and correlated with the sensitivity of HCC cells to Idarubicin.

**Conclusion:**

Lysosome-associated gene risk models built by machine learning can effectively predict patient prognosis and offer new prospects for chemotherapy and immunotherapy in HCC. In addition, cellular-level experiments suggest that RAMP3 may be a new target for the treatment of HCC.

## Introduction

Hepatocellular carcinoma is the seventh most common form of cancer and the second most common cause of cancer-related death in the world. Its incidence is on the rise and poses a serious threat to human health (1), and in China, HCC is one of the four leading causes of cancer-related death (2). There are various treatment options for HCC, such as partial hepatectomy, liver transplantation, radiofrequency ablation, hepatic artery embolization chemotherapy, and targeted therapy (3), and in recent years, as research progresses, new strategies of combining multiple chemotherapeutic agents with immunotherapy have emerged (4). Although some results have been achieved, overall, the survival benefit is very limited unless patients are stratified according to their gene expression profile (5–7). The search for more precise and effective molecular markers is therefore extremely necessary to improve clinical outcomes and reduce patient burden in patients with liver cancer.

Lysosomes are membrane-encapsulated organelles, and lysosomes were previously thought to be sites of degradation of intracellular and extracellular substances. As a result, researchers have called lysosomes the “rubbish disposals” of cells (8–10), however, more in-depth studies have shown that this view is too one-sided. Emerging evidence suggests that lysosomes may directly or indirectly regulate cell signaling, metabolism and degradation of protein aggregates and damaged organelles (11, 12). It has been shown that lysosomes may play an important role in tumor development through the above-mentioned biological processes, and that both the functional state and spatial distribution of lysosomes are closely related to cancer cell proliferation, energy metabolism, invasive metastasis, immune escape, drug resistance and tumor-associated angiogenesis (13), but there are still few reports on the relevance of lysosomes in tumors, and more importantly, we have not found any previous reports of lysosome-related genes in hepatocellular carcinoma.

The aim of this paper is to analyze the expression of lysosome-related genes in HCC and to build an optimal prognostic model based on machine learning. The features were used to stratify HCC patients by risk score. Immuno-infiltration analysis, immune checkpoint gene correlation, chemotherapy drug sensitivity, enrichment analysis and clinical relevance analysis were performed for high and low-risk groups to validate the stratification. In addition, we overexpressed RAMP3 and preliminarily demonstrated the potential of RAMP3 as a new therapeutic target by means of cell proliferation, cell migration, invasion and drug sensitivity assays. In conclusion, the present study may provide new options for the treatment and prediction of hepatocellular carcinoma.

## Materials and methods

### Data sources

The mRNA sequencing data and corresponding clinical information (TCGA-LIHC) for hepatocellular carcinoma were obtained from TCGA (https://portal.gdc.cancer.gov/), which included 374 liver cancer samples and 50 normal tissue samples; and from the GEO database (https://www.ncbi.nlm.nih.gov/geo/) to obtain the hepatocellular carcinoma-related dataset GSE14520, based on the GPL3921 platform (Affymetrix HT Human Genome U133A Array), containing 225 hepatocellular carcinoma samples and 220 normal samples; lysosomal-related genes (875) were obtained from AmiGO2 (http://amigo.geneontology.org/amigo) was obtained. Data were processed using R (4.2.0).

### Differential gene analysis

The “edgeR” package (14) was used to identify genes differentially expressed in TCGA-LIHC in liver cancer samples and normal tissues; the Sanger assistant (15) was used to take intersections for differential genes and lysosome-related genes; the “corrplot” package and “tinyarray” package were used to plot correlations as well as heat maps.

### Gene function analysis

Enrichment analysis was performed using the online website DAVID (https://david.ncifcrf.gov/tools.jsp) and P values less than 0.05 were considered significant and visualized by the Sanger assistant. Gene expression in single cells of hepatocellular carcinoma was analyzed using the single cell database TISCH (http://tisch.comp-genomics.org/search-gene/).

### Machine learning

The liver cancer samples from TCGA-LIHC were filtered (filtering criteria: remove samples with incomplete survival information or survival time less than 30 days), and finally 343 liver cancer samples were obtained from TCGA-LIHC (training set); clinical information of GSE14520 was obtained from (16), which was also filtered (filtering criteria: remove samples with incomplete survival information or survival time less than 30 days), and finally 343 liver cancer samples were obtained from GSE14520 (training set); clinical information of GSE14520 was also filtered (filtering criteria: remove samples with incomplete survival information or survival time less than 30 days). samples), resulting in 219 columns of liver cancer samples from GSE14520 (test set).

A preliminary screen for differential lysosomal-associated genes in hepatocellular carcinoma was performed using a univariate Cox (“survival” package) to derive lysosomal-associated genes associated with overall survival (OS) for machine learning. Random forest (RSF) analysis was performed using the “randomForest” package to select (the top 8 ‘significant’ genes for subsequent analysis); Lasso analysis was performed using the “glmnet” package, with the optimal value of the penalty paraeter (λ) determined based on a ten-fold cross-validation used to select significant features; Stepwise regression (stepwise) using the My. “Stepwise” package. The algorithms were evaluated by combining the three algorithms in pairs or individually on the training set, with the average C-index value of the training and test sets.

### Building the model

The signature was constructed using COX regression to construct a risk model based on the following equation


riskscore=(0.173*CD68)+(−0.359*RAMP3)+(0.193*CSPG5)+(0.0657*FABP5)+(0.0276*CSF3R)+(0.189*CSPG4)+(−0.0434*PLD1)+(0.0792*SORT1)

### Assessment model

Hepatocellular carcinoma samples were divided into high and low-risk groups based on median risk and the effect of the model was assessed using C-index,K-M,ROC.

Explore differences in biology, immune microenvironment, immunotherapy and tumour chemotherapy sensitivity between high and low-risk groups

The GSVA package and “msigdbr” package were used to explore the functional differences in biology between the high and low-risk groups. The reference gene set for KEGG analysis was species = “Homo sapiens”, category = “C2”, subcategory = “CP: KEGG”; the reference gene set for GO analysis was species = Homo sapiens, category = “C5”.

The ssGSEA function in the “GSVA” package was used to calculate the abundance of 28 immune cells in liver cancer samples; the “IMvigor210CoreBiologies” package (17) was used to predict the responsiveness of high and low-risk groups to immunotherapy.

Common anticancer drug sensitivities between high and low-risk groups were predicted using the “Prrophetic” package (18) based on matrix padding and ridge regression models.

### Cell culture

Human HCC cell lines (Huh7, HepG2, SNU387, MHCC97H, Hep3B) were purchased from the Shanghai Cell Collection, Chinese Academy of Sciences. Cells were cultured in an incubator supplemented with 10% fetal bovine serum (Gibco, Grand Island, USA), 100 U/mL penicillin and 100 mg/mL streptomycin (Gibco, Grand Island, USA) at 37°C and 5% CO2.

### Transfection

The plasmid was purchased from (GenePharma Co. Ltd., Shanghai, China). Transfection reagent was purchased from (Thermo Fisher Scientific, Shanghai, China). After the cells have reached 60-70% growth, Lipofectamin 3000 was added to 100 µl of serum-free medium, and the pcDNA and P3000 (1:1) were added to 100 µl of serum-free medium, both were mixed and incubated for 15 min at room temperature. After incubation, the mixture is added to each well (12-well plate) with 800 ul of serum-free medium and then 200 ul of P3000-Lipofectamin3000-pcDNA mixture is added to each well and incubation is continued at 37°C in a constant temperature incubator; after 24-36 h of transfection, subsequent experiments can be carried out.

### CCK-8 assay

A 96-well plate with 5000 cells per well was used and 5 replicate wells were set up. CCK-8 reagent (BioSharp, Beijing, China) was added at 0h, 24h, 48h and 72h for detection in an enzyme marker, and cell growth curves were plotted using mapping software and analysed for statistical significance.

### Transwell migration

Matrigel (Corning,Shanghai,China) matrix gel was added to the small chambers in advance and allowed to solidify. 200 µl of cell suspension (5x10^5^ cells) was added to the upper chamber and 800 µl of complete medium containing 10% FBS was added to the 24-well plate (lower chamber). The 24-well plates were placed in an incubator and incubated for 48h before fixed staining. The cell migration assay is performed as the cell invasion assay, except that no matrix gel is required in the upper chamber and incubated for 36 hours.

### Transwell invasion

The same as migration except that no matrix gel is added to the upper chamber.

### RT-PCR

Total RNA was extracted using Total RNA Kit I (OMEGA biotek, USA) and complementary DNA (cDNA) was synthesised using a reverse transcription kit (Thermo Fisher Scientific, Waltham, Massachusetts, EUA). The primers used for the quantitative real-time PCR (GenePharma Co. Ltd., Shanghai, China) were as follows: RAMP3 (5’-GGCATCCACAGGCAGTTCTT -3’ and 5’-CGGGTATAACGATCAGCGGG-3’); β-actin (5’-GAG AAA TCT GGC ACC ACA CC-3’ and 5’-GGA TAG CAC AGC CTG GAT AGCAA-3’).

### Western blotting

Equal amounts of protein extracts were separated by SDS-PAGE and transferred to PVDF membranes using antibodies against RAMP3 (R&D Systems, Shanghai, China) and Tubulin (Abcam, Shanghai, China). The signals were detected using the Immobilon western chemilum HRP Substrate (BioSharp, Beijing, China), and images were obtained by a GEL-DOC2000 Gel Imager system (BIO-RAD, California, USA).

### Data analysis

Statistical analyses were performed using GraphPad Prism (8.0.2) and R software (4.2.0). P < 0.05 was considered statistically significant.

## Results

### Identification of differential lysosome-associated genes in hepatocellular carcinoma

**Figure 1:**
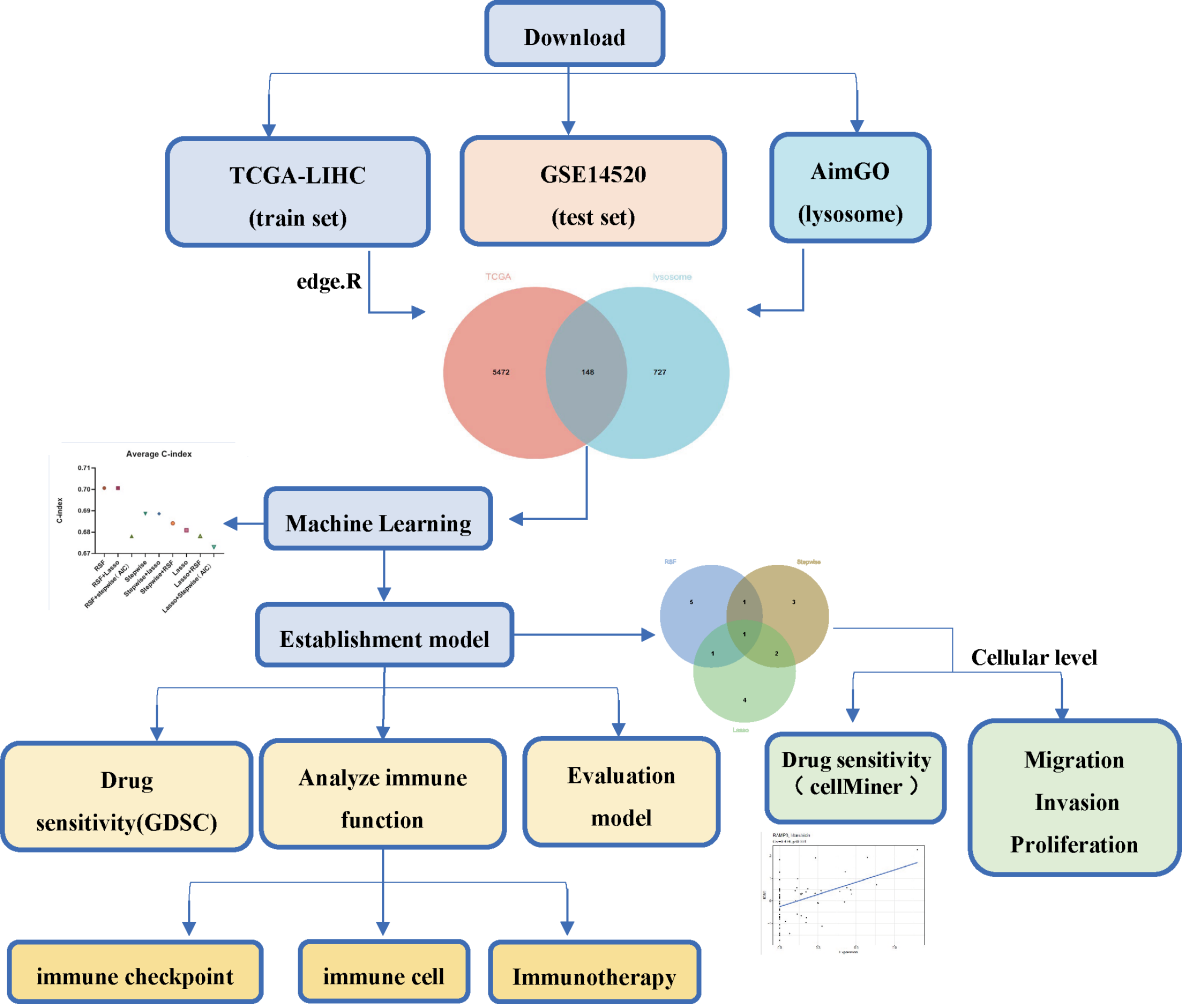
The workflow of the current study.

**Figure 2:**
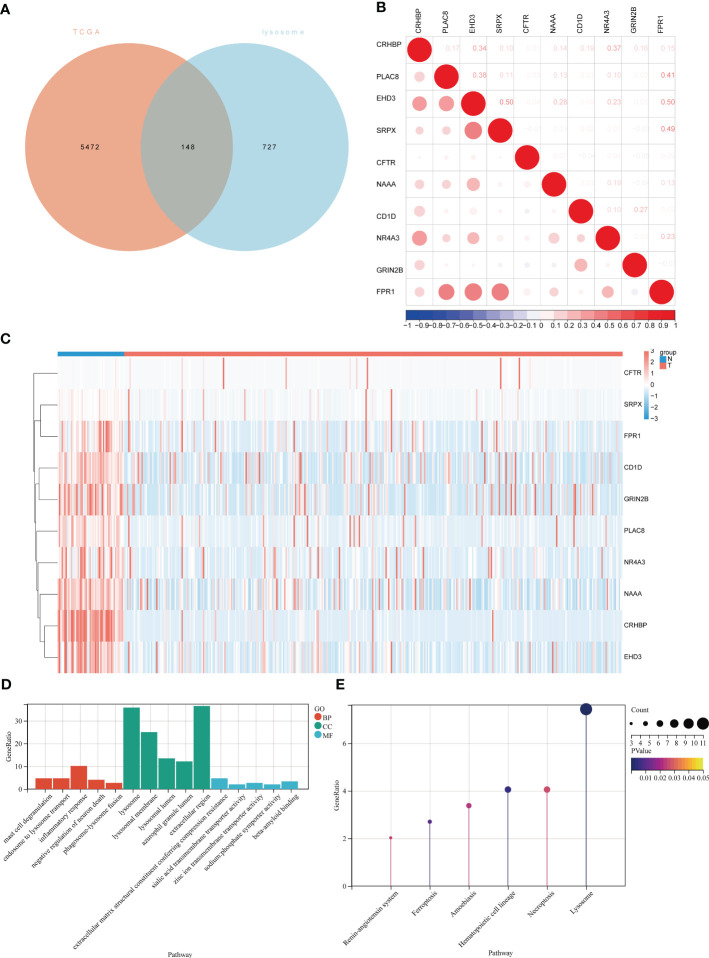
Expression of lysosome-related genes in hepatocellular carcinoma and normal tissues and demonstration of the biological functions involved. **(A)**Venn diagram of intersection of differentially expressed genes in TCGA-LIHC and lysosomal genes in AIMGO; **(B)** scatter plot showing the correlation between the top 10 differentially expressed lysosome-related genes; **(C)** hierarchical clustering of the top 10 differentially expressed lysosome-related genes. Blue bars represent normal tissues and red bars represent liver cancer tissues. **(D)** GO results of lysosome-related genes differentially expressed in liver cancer tissues and normal tissues, red bars represent Biological Process, green bars represent Cellular Component, blue bars represent Molecular Function; **(E)** KEGG results of lysosome-related genes differentially expressed in liver cancer tissues and normal tissues.

The flowchart of the current study is shown in **Figure 1**. To identify differential lysosome-associated genes in hepatocellular carcinoma (HCC), we obtained 374 HCC samples and 50 normal tissue samples from TCGA and performed differential analysis using the edge package (threshold value for differential genes |logFC | ≥ 1, p-value < 0.05). This analysis revealed 5620 genes (2593 up-regulated and 3027 down-regulated) that were differentially expressed in HCC and normal tissue (**Additional file 1: Table S1**). We then intersected these results with 875 lysosome-associated genes from AmiGO2 (**Additional file 1: Table S2**) to obtain 148 genes (**Figure 2A**). The top 10 differential genes were subjected to correlation analysis (**Figure 2B**), and a heat map was generated to visualize their expression patterns (**Figure 2C**). Gene Ontology (GO) and Kyoto Encyclopedia of Genes and Genomes (KEGG) enrichment analysis (**Figures 2D, E**) revealed that differentially expressed lysosome-related genes were enriched in pathways associated with tumor progression, including Lysosome, Ferroptosis, Necroptosis, and inflammatory response. In summary, we identified 148 differential lysosome-associated genes in HCC and found that they were enriched in pathways associated with tumor progression. These results provide insight into the role of lysosomes in HCC and lay the foundation for further analysis and experimentation.

### Model construction by machine learning

**Figure 3:**
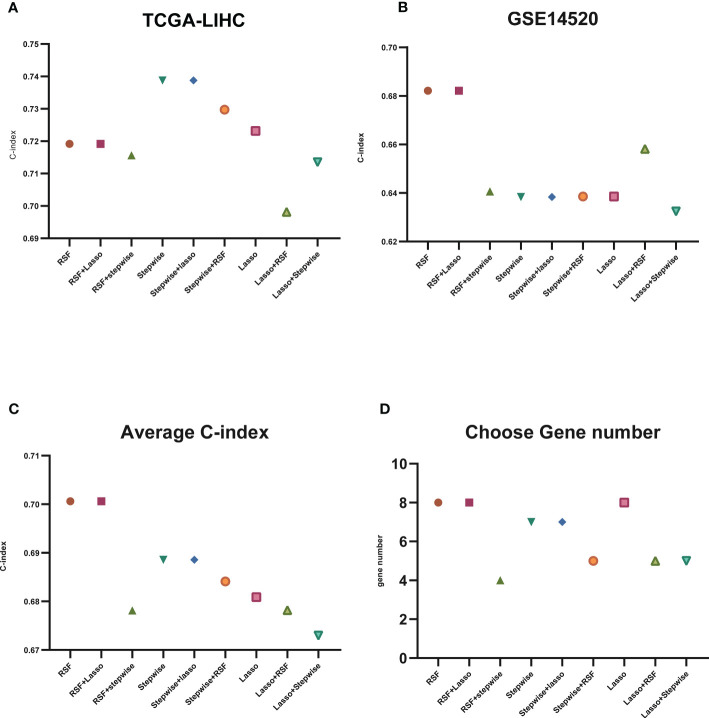
C-index display of machine learning. **(A)** C-index display of the nine algorithms in TCGA-LIHC (training set); **(B)** C-index display of the nine algorithms in GSE14520 (test set); **(C)** average C-index of TCGA-LIHC (training set) and GSE14520 (test set); **(D)** nine algorithms selected for the number of genes.

To construct a model using machine learning, we first screened 148 lysosome-related genes using univariate COX analysis with a significance threshold of P<0.05. This resulted in 46 genes with prognostic significance, which were used for further analysis. A common method for constructing models in previous studies was the Lasso method (19, 20). In previous studies, the Lasso method was commonly used to construct models, but we found that this may not be the best approach for our data (21). Therefore, we chose three common ways of constructing models (RSF, Lasso, stepwise) (20, 22, 23) either separately or in two-by-two combinations to analyze the 46 lysosomal-associated genes.We calculated the C-index for both the training set (TCGA) and the test set (GSE14520) separately and averaged the results (**Figures 3A–C**; **Additional file 2: Figures S1**-**S3**). We also counted the number of genes used in the nine algorithms that were combined to construct the model (**Figure 3D**).

**Figure 4:**
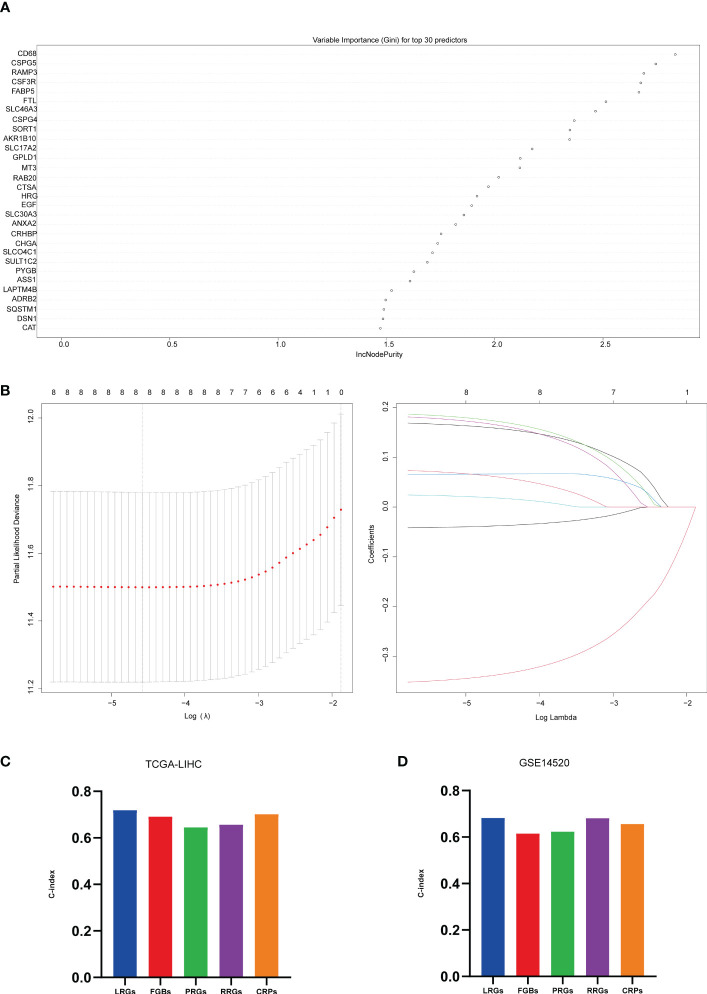
Machine learning constructed models. **(A)** top 30 significant genes screened by random forest in the training set; **(B)** further screening of the top 8 significant genes screened by random forest by Lasso, λ = lambda.min; **(C)** C-index of 5 prognostic models in TCGA-LIHC; **(D)** C-index of 5 prognostic models in GSE14520.

The results of our study indicate that some algorithms, such as stepwise and Lasso, performed well in the training set but did not perform well in the test set. Therefore, we selected the genes identified by RSF+Lasso to construct our model based on the combined performance of the algorithms. (**Figures 4A, B**; **Additional file 2: Figures S2C, D**). To test the predictive power of our model (LGRs), we compared it with four published prognostic models, including FGBs (Five-Gene-Based Prognostic Signature) (24), PRGs (Pyroptosis-Related Gene Signature) (25), RRGs (Response-Related Gene Signature) (26), and CRGs (Cuproptosis-Related Gene Signature) (27). We used C-index to evaluate the predictive ability of the model, and our analysis showed that our model had the highest C-index in both the training and test sets (**Figures 4C, D**). In conclusion, our constructed prognostic models based on lysosome-related genes (LGRs) have superior performance compared with published models.

### Evaluating the model

**Figure 5:**
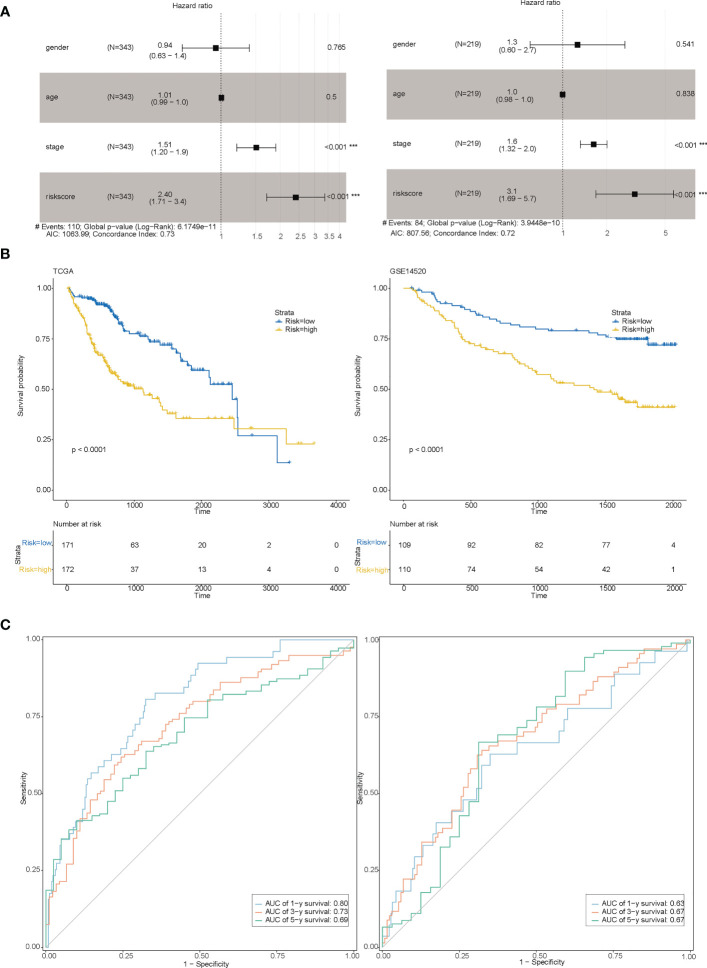
Evaluation of the model. **(A)** Multi-factor COX for both the training set (Figure left) and the test set (Figure right) indicating that risk scores are associated with prognosis; **(B)** Survival curves between the high-risk and low-risk groups for both the training set (Figure left) and the test set (Figure right); **(C)** 1,3,5-year RCO curves between the high-risk and low-risk groups for both the training set (Figure left) and the test set (Figure right).

After constructing the risk model, we categorized the sample into high and low-risk based on the median risk value and found that the eight genes (genes) used to construct the model were mostly differentially expressed between the high and low-risk groups (**Additional file 3: Figures S4A, B**). Risk factor plots (**Additional file 3: Figures S4C, D**) showed that risk scores were negatively associated with overall survival and survival status of patients. Combining the risk score with clinical information from other liver cancer samples in a multifactorial COX (**Figure 5A**) showed that the risk score was indeed an independent prognostic factor for patients with liver cancer and that the C-index at this point was ≥0.72. Based on the risk grouping, we then performed a Kaplan-Meier (KM) survival analysis (**Figure 5B**), which showed that the high-risk group had a poorer prognosis. In addition, we plotted the corresponding ROC curves for each group using years 1,3,5 as the endpoints of prediction time (**Figure 5C**), and the results demonstrated good predictive power (AUC ≥ 0.69 in the training set; AUC ≥ 0.63 in the test set).

### Exploring biological function between high-risk and low-risk groups

**Figure 6:**
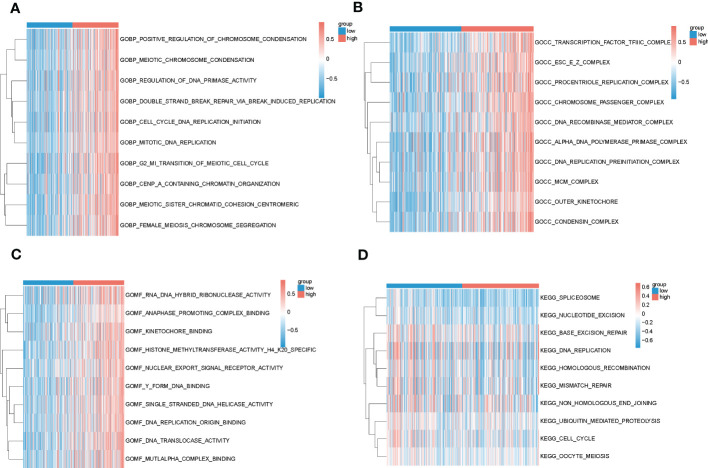
Results of GSVA analysis of high and low-risk groups in TCGA. **(A–C)** Biological processes, cellular localization and molecular function enrichment pathways in GO for high and low-risk groups, blue for low-risk group and red for high-risk group; **(D)** Pathways enriched in KEGG for high and low-risk groups, blue for low-risk group and red for high-risk group.

In order to gain insights into the biological mechanisms underlying the differences between high-risk and low-risk groups, we performed enrichment analysis using the “GSVA” and “msigdbr” packages. The top 10 pathways that emerged from our analysis were visualized in **Figures 6A–D**. Our results suggest that the differential genes between these two groups are primarily involved in the SPLICEOSOME, CELL_CYCLE, and DNA_REPLICATION pathways. These findings provide important clues for further investigation into the mechanisms underlying the development and progression of high-risk cancers.

### Relationship between risk grouping and immune microenvironment of liver cancer and immunotherapy

**Figure 7:**
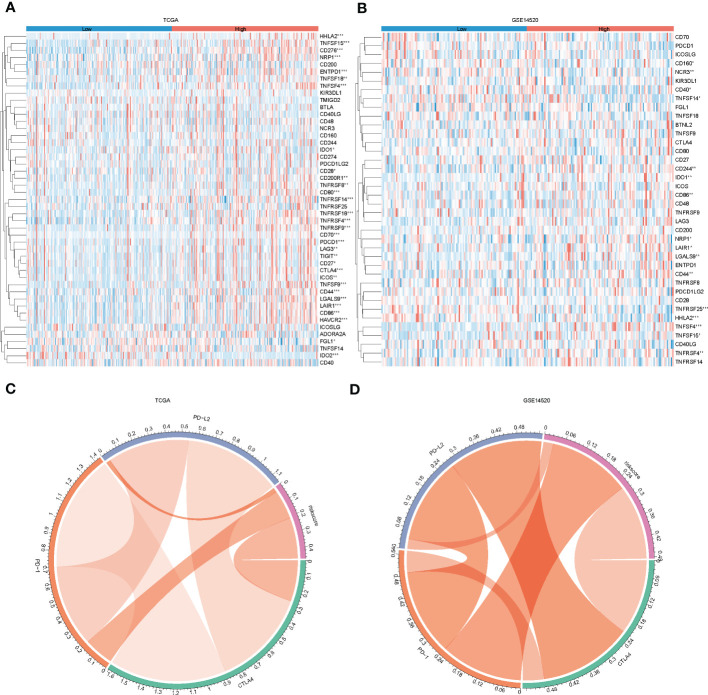
Immune checkpoint differences between high and low-risk groups. **(A, B)**, heat map showing immune checkpoint differences between high and low-risk groups in the training and test sets; **(C, D)** chord plot showing correlations between risk scores and PD-L2, PD-1 and CTLA4 in the training and test sets.

In recent years, the success of immune checkpoint therapy has highlighted the crucial role of the immune system in cancer treatment (28). Lysosomes have been identified as a major site for the degradation of immune checkpoint molecules, as they can temporarily store proteins such as CTLA-4, PD-L1, TIM-3, CD70, CD200, and CD47 (29). Therefore, we analyzed the expression levels of immune checkpoints (27) in the high and low-risk groups in both the training and test sets (**Figures 7A, B**). We also visualized the correlation between riskscore and PD-1, CTLA-4, and PD-L1 (**Figures 7C, D**). Our results indicate that CD244, CD44, and TNFRSF14 (P < 0.05) were significantly different between the high and low-risk groups. These findings suggest that immune checkpoint molecules may play an important role in the development and progression of high-risk cancers, and may be potential targets for cancer immunotherapy.

**Figure 8:**
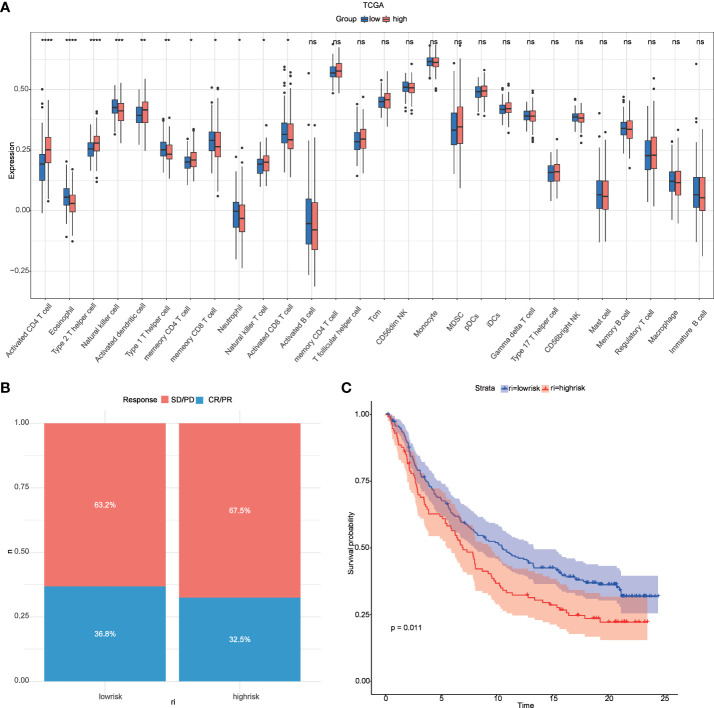
Relevance of risk scores to immune cells and to immunotherapy. **(A)** GSVA analysis of immune cell differences between high and low-risk groups in TCGA; **(B)** distribution of CR/PR and SD/PD between high and low-risk groups in the immunotherapy cohort; **(C)** survival curves between high and low-risk groups in the immunotherapy cohort. *P < 0.05 **P < 0.01, ***P < 0.001, ****P < 0.0001. ns, no significance.

According to the report lysosomes can also be involved in the regulation of immune cell function (30), so we calculated the abundance of immune cells in liver cancer samples by the ssgsea function in the GSVA package , and the box plot (**Figure 8A**) both demonstrate the difference in immune cells between high and low-risk groups, and the results show that there are natural killer cells(NK), T helper 2 cell(Th2), T helper 1 cell(Th2), and Natural killer T (NKT) cells between high-risk and low-risk groups differential expression *(P < 0.05)*, which is the same as that reported in the literature (31). In addition, we compared the responsiveness of the high-risk and low-risk groups to immunotherapy (32, 33) and found that patients in the low-risk group responded better to immunotherapy than those in the high-risk group (**Figure 8B**) and that patients in the low-risk group had a better prognosis than those in the high-risk group (**Figure 8C**).

### Differences in drug sensitivity between high–risk and low-risk groups

**Figure 9:**
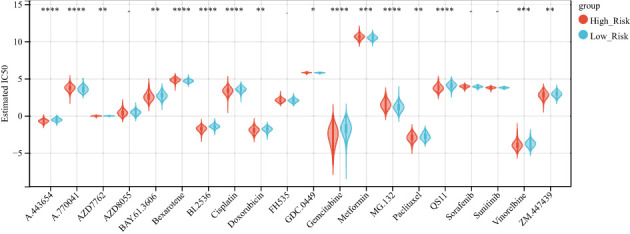
high and low-risk groups are associated with multiple chemotherapy drug sensitivities. Boxplot showing the difference in chemotherapy drug sensitivity between high and low-risk groups, with red representing the high-risk group and blue representing the low-risk group. *P < 0.05 **P < 0.01, ***P < 0.001, ****P < 0.0001.

The emergence of drug resistance has greatly reduced the therapeutic efficacy of oncological chemotherapeutic agents, and the lysosome has recently emerged as a promising target for overcoming chemotherapy resistance (33), and evidence suggests that interfering with lysosomal function may be a way in which chemotherapy can be sensitized, an effect that may arise by affecting multiple mechanisms, such as trafficking in the FEFFLUX transporter, drug sequestration and TFEB-regulated pathways that including autophagy and DNA repair (34). Therefore, we used the GDSC database to predict the sensitivity of 20 commonly used hepatocellular carcinoma drugs in high- and low-risk groups (**Additional file 4: Table S3**), and there was a difference in the sensitivity of 16 hepatocellular carcinoma drugs between high- and low-risk groups (**Figure 9**).

### Single cell analysis

**Figure 10:**
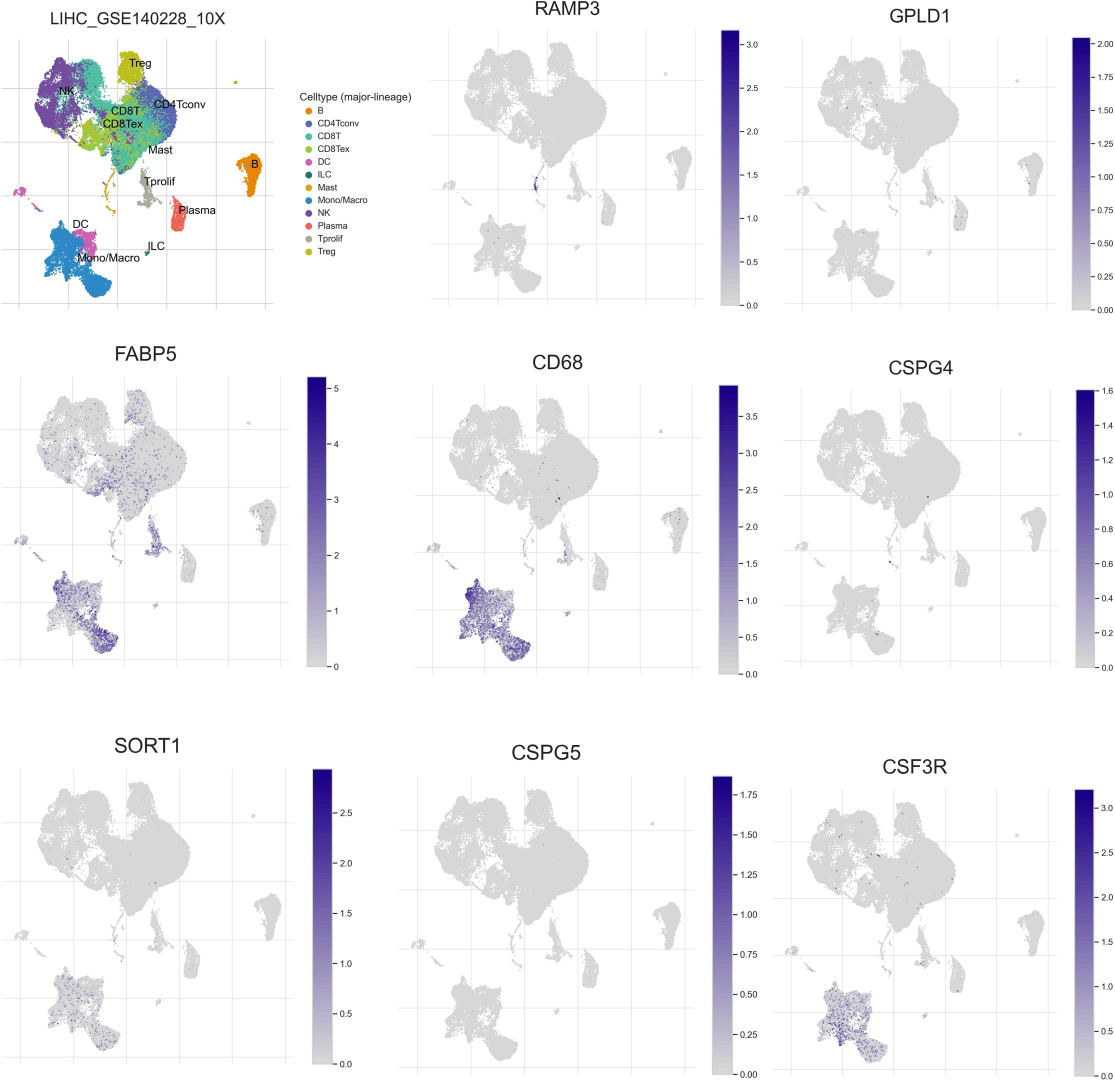
Expression of the eight constructed model genes at the single cell level.

To investigate the expression patterns of 8 genes (RAMP3, GPLD1, FABP5, CD68, CSPG4, SORT1, CSPG5, CSF3R) in various immune cell subpopulations of hepatocellular carcinoma, we utilized the single-cell database TISCH (http://tisch.comp-genomics.org/search-gene/) to analyze GSE140228, which consisted of 62,530 cells. The results depicted in **Figure 10** indicated that FABP5, CD68, SORT1, and CSF3R were predominantly expressed in monocytes/macrophages, while RAMP3 was primarily expressed in Treg cells. GPLD1, SORT1, and CSPG5 showed low levels of expression in GSE140228.

### RAMP3 is associated with proliferative capacity, migratory and invasive capacity and drug sensitivity of hepatocellular carcinoma cells

To identify key genes related to lysosomes, three basic algorithms (RSF, Lasso, and stepwise) were used, and RAMP3 was selected for further study based on the results. The expression of RAMP3 in tumor cell lines and normal cell lines was explored in BioGPS (http://biogps.org/)(**Additional file 5: Figures S5A, B**).

It has been shown that lysosomes are involved in regulating the proliferation, migration and invasion of tumour cells (35, 36). We hypothesized that RAMP3 might also be associated with the proliferation, migration and invasive ability of hepatocellular carcinoma cells. RAMP3 expression was detected by RT-PCR in five common laboratory hepatocellular carcinoma cell lines (Huh7, HepG2, SNU387, MHCC97H and Hep3B) (**Additional file 5: Figure S5C**), followed by overexpression of RAMP3 in Hep3B and HepG2 (**Additional file 5: Figures S5D, E**), followed by separate CCK-8 proliferation, transwell proliferation, migration and invasion assays on hepatocellular carcinoma cells. The results showed that the proliferation; migration and invasion abilities were significantly reduced in the RAMP3 overexpression group compared to the control group (**Additional file 5: Figures S5F–H**).

Furthermore, analysis of the relationship between RAMP3 and chemotherapeutic drug sensitivity through the CellMiner database (https://discover.nci.nih.gov/cellminer/home.do) (37) (**Additional file 6: Figure S6**) showed that RAMP3 expression correlated with multiple chemotherapeutic drugs (P < 0.05), with the highest correlation being with Idarubicin (R = 0.474, P < 0.001). Idarubicin is not only the most toxic drug to human hepatocellular carcinoma cell lines, but also has the ability to overcome multidrug resistance (38, 39), suggesting to us the possibility of RAMP3 being a drug target.

## Discussion

It is estimated that every year, around 841,000 new cases of hepatocellular carcinoma (HCC) are diagnosed, with 781,631 patients dying from the disease in 2018 alone (40). Despite advancements in early detection and drug development, the clinical outcomes for advanced cases of HCC remain unsatisfactory. Therefore, there is an urgent need to identify new and effective molecular markers to improve clinical outcomes and reduce the burden of HCC cases (38, 41).

Lysosomes are an important component of the inner membrane system and participate in numerous cell biological processes, such as macromolecular degradation, antigen presentation, intracellular pathogen destruction, plasmamembrane repair, exosome release, cell adhesion/migration and apoptosis (42). Recent studies have shown that the functional state and distribution of lysosomes also regulate tumour development and progression. However, there are still few reports on lysosomes in HCC. To address this gap, we developed a prognostic model of lysosome-related genes using a machine learning approach. We also investigated the relationship between these genes and the immune microenvironment, immunotherapy, and drug sensitivity. This research has the potential to contribute to the development of new HCC treatments and improve patient outcomes.

In this study, a machine learning approach was used to construct a prognostic risk model consisting of eight genes (RAMP3, GPLD1, FABP5, CD68, CSPG4, SORT1, CSPG5, CSF3R). Several of these genes have been previously linked to cancer, such as RAMP3, which has been shown to inhibit the metastatic ability of liver cancer cells when lacking in cancer fibroblasts. Targeting GPLD1 has been found to inhibit the proliferation of non-small cell lung cancer cells mediated by p38 MAP kinase (43) Knockdown or silencing of FABP5 has been shown to reduce the proliferation and invasiveness of PC cells *in vitro* and reduce tumor growth and metastasis *in vivo* (44). Additionally, hsa_circ_0110389 has been found to upregulate SORT1 to promote gastric cancer progression by sponging miR-127-5p and miR-136-5p (45). The risk model was evaluated in both training and test set samples by dividing the samples into two groups based on the median value of risk. Patients in the low-risk group had significantly longer survival, and the ROC curves validated the predictive validity of the risk score. Multifactorial COX demonstrated that the risk model was an independent prognostic factor for liver cancer. Subsequent analysis of the functional differences between the high and low-risk groups in TCGA using GSVA showed that the differential genes between the two groups were mainly involved in the SPLICEOSOME, CELL_CYCLE, and DNA_REPLICATION pathways.

In recent years, the use of immune checkpoint inhibitors (ICIs) has revolutionized cancer treatment. However, many patients with hepatocellular carcinoma (HCC) still do not respond well to ICBs (46). Research has shown that lysosomes can be a primary site for the degradation of immune checkpoint molecules. Therefore, we investigated the relationship between risk models and immune checkpoints and found significant differences in CD244, CD44 TNFRSF14, CD27, and other immune checkpoints between high- and low-risk groups. The infiltration of immune cells is a critical factor in the prognosis of HCC patients (47). Tumor infiltration and the recurrence of circulating NK cells are positively associated with survival benefits in HCC with prognostic significance (48). Our results showed that NK cell levels were lower in the low-risk group than in the high-risk group, which is consistent with previous studies (31). Moreover, our analysis revealed that the low-risk group had better results for immunotherapy and a more favorable prognosis than the high-risk group in the immunotherapy cohort. Additionally, risk scores were associated with multiple chemotherapeutic drug sensitivities.

RAMP3 has been selected by multiple algorithms and ranked highly in random forests; therefore, we believe that RAMP3 is a key gene in lysosomal-related genes and that RAMP3 has not been studied in hepatocellular carcinoma. We demonstrated at the cellular level that overexpression of RAMP3 significantly reduced the proliferation, migration and invasion of hepatocellular carcinoma cells. Furthermore, we found that RAMP3 was associated with idarubicin, which has been shown to improve remission rates in intermediate stage hepatocellular carcinoma (49), suggesting the possibility that RAMP3, like other small molecule drugs (50) being investigated, could be a new drug target.

Unfortunately, our study has some limitations. First, although the predictive power of our model is better than some published prediction models, the predictive power of LRGs is still inadequate compared to some machine learning constructed prognostic models (51, 52) for liver cancer. Second, further experiments are needed to explore the pathological functions of the other seven lysosome-related genes in HCC. Third, although we have demonstrated that RAMP3 can inhibit the proliferation, migration and invasion of hepatocellular carcinoma cells, the underlying mechanisms need to be further investigated *in vivo*. The above deficiencies will be the focus of our future work.

## Conclusion

Our study identifies a prognostic signature based on eight lysosome-related genes and this model not only predicts patient response to immunotherapy and chemotherapeutic agents, but also has high accuracy in predicting overall patient survival. Furthermore, we demonstrated at the cellular level that RAMP3 correlates with the proliferation, migration, and invasive ability of hepatocellular carcinoma cells.

## Data availability statement

The datasets presented in this study can be found in online repositories. The names of the repository/repositories and accession number(s) can be found within the article/**Supplementary Materials**.

## Author contributions

WL.: Create study concept, statistical analysis, analyze and interpret data, and write the manuscript. QW: Perform cell culture, WB and PCR. JL, BZ and YG: Assist in WB and cell experiments. XW: Interpret data and provide technical or material support. XC: Propose project concepts, supervise projects and experiments, interpret data, and write manuscripts. All authors contributed to the article and approved the submitted version.

## Supplementary material

Supplementary Table 1 Genes from TCGA that are differentially expressed in liver cancer tissue and normal tissue.

Supplementary Table 2 Set of lysosome-related genes from AmiGO2.

Supplementary Table 3 IC50 prediction between high risk and low risk groups.

Supplementary Figure 1 C-index of Lasso, Lasso+RSF and Lasso+stepwise algorithms. **(A)** C-index of the prognostic model constructed based on Lasso (TCGA on the left side of the figure, GSE14520 on the right side of the figure). **(B)** C-index of the prognostic model based on Lasso+RSF (TCGA on the left side of the figure and GSE14520 on the right side of the figure). **(C)** C-index of the prognostic model based on Lasso+stepwise construction (TCGA on the left, GSE14520 on the right).

Supplementary Figure 2 C-index of RSF, RSF+Lasso and RSF+stepwise algorithms are shown. **(A)** C-index of the prognostic model constructed based on RSF (TCGA on the left of the figure, GSE14520 on the right of the figure). **(B)** C-index of the prognostic model constructed based on RSF+Lasso (TCGA on the left, GSE14520 on the right). **(C)** C-index of the prognostic model constructed based on Lasso+stepwise (TCGA on the left and GSE14520 on the right).

Supplementary Figure 3 C-index demonstrations for the 3 algorithms stepwise, stepwise+Lasso and stepwise+RSF. **(A)** C-index of the stepwise based prognostic model (TCGA on the left and GSE14520 on the right). **(B)** C-index of the prognostic model based on stepwise+Lasso (TCGA on the left, GSE14520 on the right). **(C)** C-index of the prognostic model constructed based on stepwise+RSF (TCGA on the left, GSE14520 on the right).

Supplementary Figure 4 Gene expression between high and low risk groups. **(A)** Differences between high and low risk groups of 8 genes in TCGA **(B)** Differences between high and low risk groups of 8 genes in GSE14520 **(C)** Risk factor plot demonstrating the relationship between gene expression and patient survival in TCGA. **(D)** Risk factor plot showing the relationship between gene expression and patient survival in GSE14520, with blue representing the low risk group and red representing the high risk group.

Supplementary Figure 5 Overexpression of RAMP3 inhibits the proliferation, migration and invasion of hepatocellular carcinoma cells. **(A, B)** Expression of RAMP3 in normal and tumor cells (BioGPS). **(C)** RT-PCR to detect the expression of RAMP3 in five hepatocellular carcinoma cell lines. **(D, E)** RT-PCR and Western Blot to detect the overexpression of RAMP3 in HepG2 and Hep3B cells **(F)** CCK-8 curves demonstrating the proliferation of overexpressed RAMP3 and control hepatocellular carcinoma cells **(G, H)** transwell to detect the migration and invasion of overexpressed RAMP3 and control hepatocellular carcinoma cells, the following figure shows the statistics.

Supplementary Figure 6 Correlation analysis of RAMP3 expression with multiple chemotherapeutic agents in cellMiner database.
