# Supplementary figures and images for: Machine learning-based prognostic modeling of lysosome-related genes for predicting prognosis and immune status of patients with hepatocellular carcinoma
Source: Front Immunol. 2023 May 19;14:1169256. doi: 10.3389/fimmu.2023.1169256 (PMC10237352; doi:10.3389/fimmu.2023.1169256)

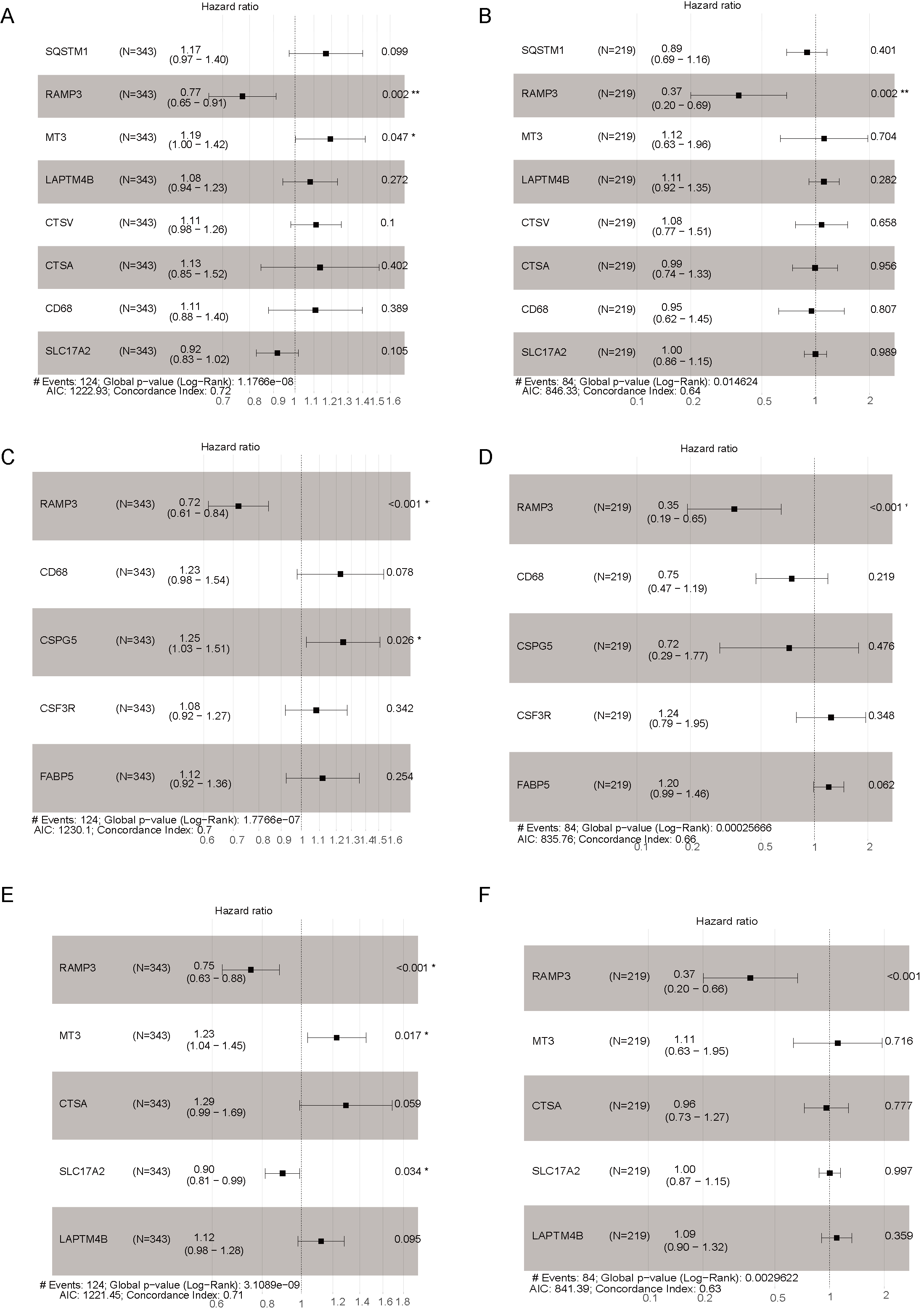

Supplement: Supplementary Table 1 — Genes from TCGA that are differentially expressed in liver cancer tissue and normal tissue. [file DataSheet_1.zip › supplemengtary material/Additional file 2/figs1.tif]

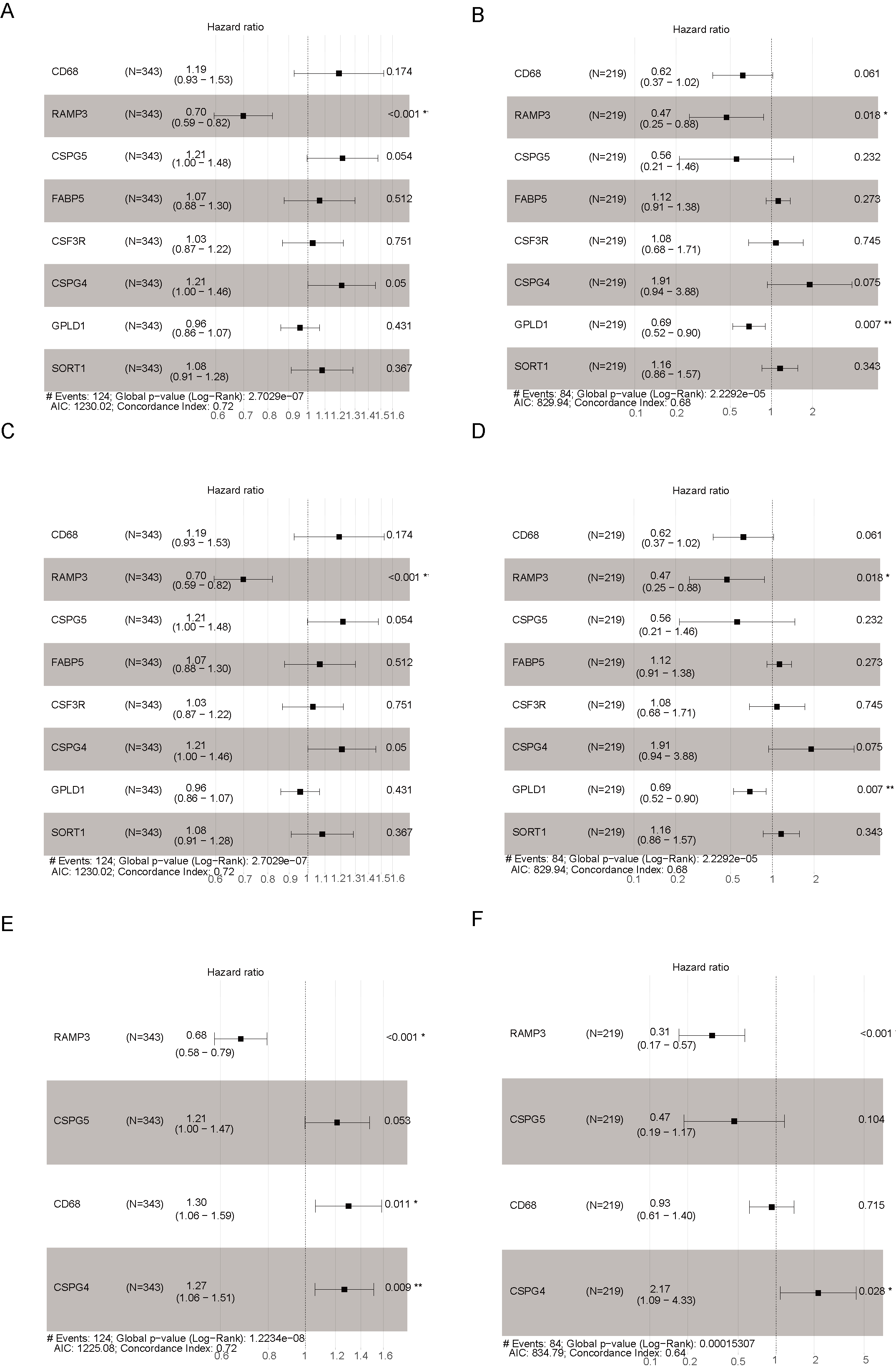

Supplement: Supplementary Table 1 — Genes from TCGA that are differentially expressed in liver cancer tissue and normal tissue. [file DataSheet_1.zip › supplemengtary material/Additional file 2/figs2.tif]

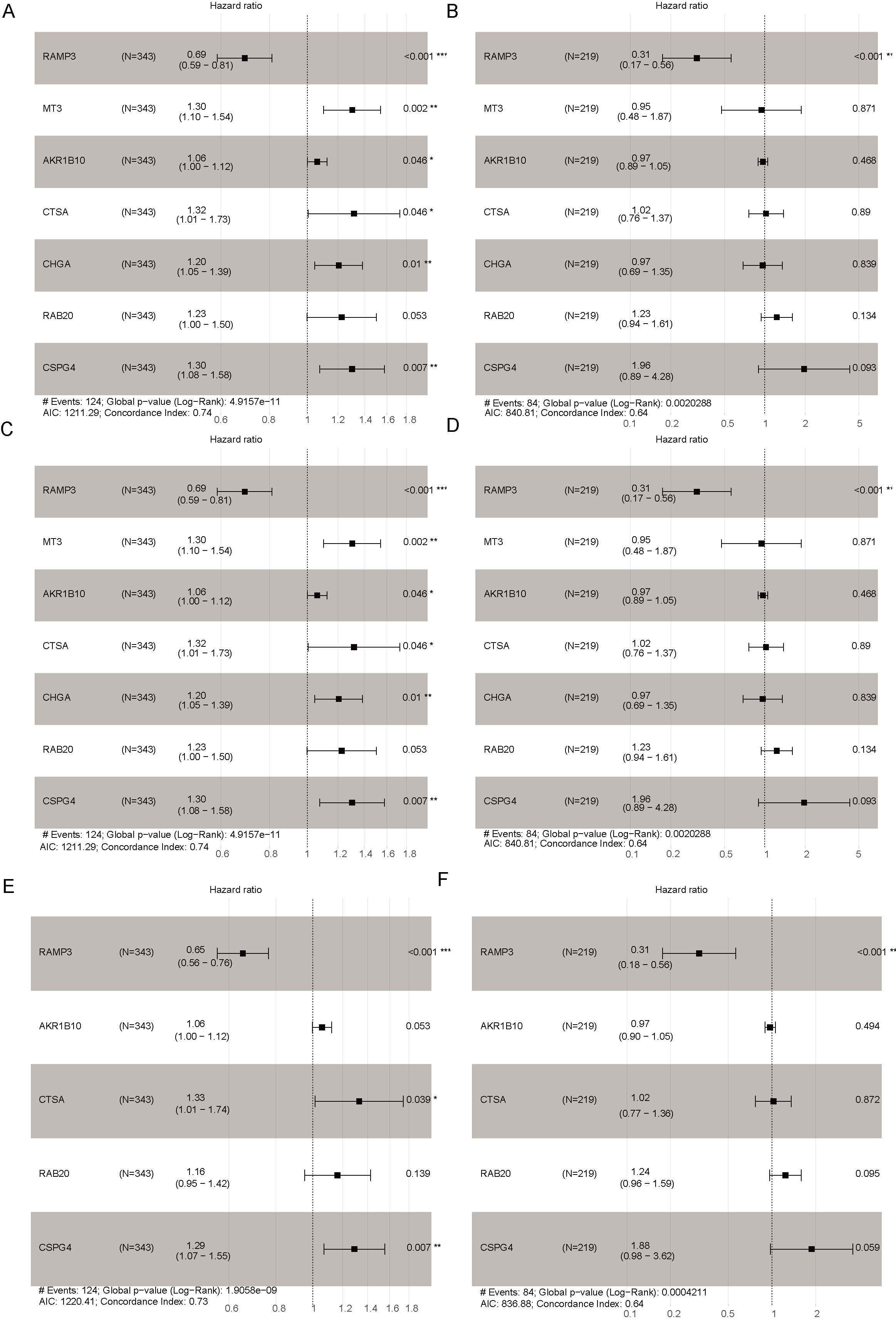

Supplement: Supplementary Table 1 — Genes from TCGA that are differentially expressed in liver cancer tissue and normal tissue. [file DataSheet_1.zip › supplemengtary material/Additional file 2/figs3.tif]

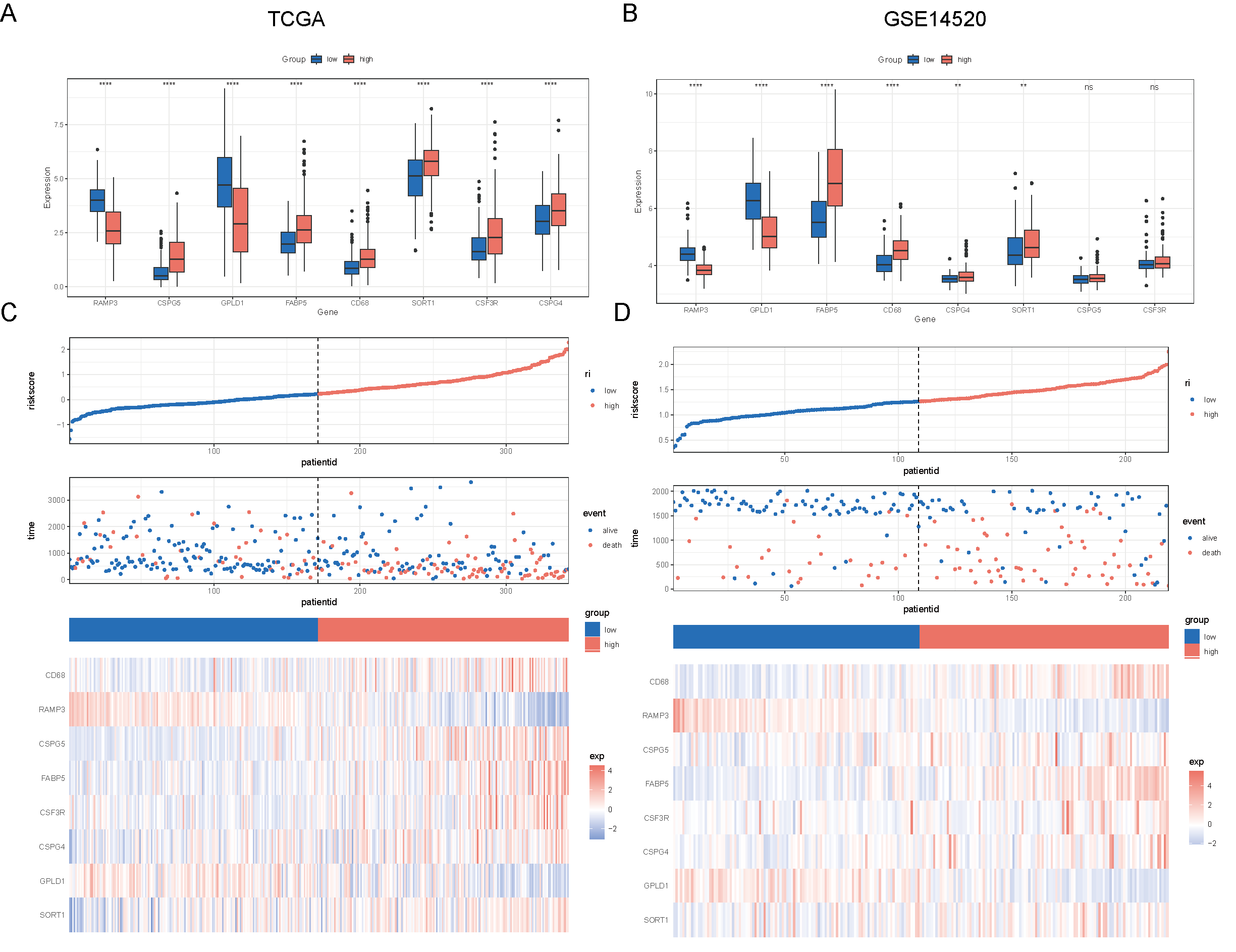

Supplement: Supplementary Table 1 — Genes from TCGA that are differentially expressed in liver cancer tissue and normal tissue. [file DataSheet_1.zip › supplemengtary material/Additional file 3/figs4.tif]

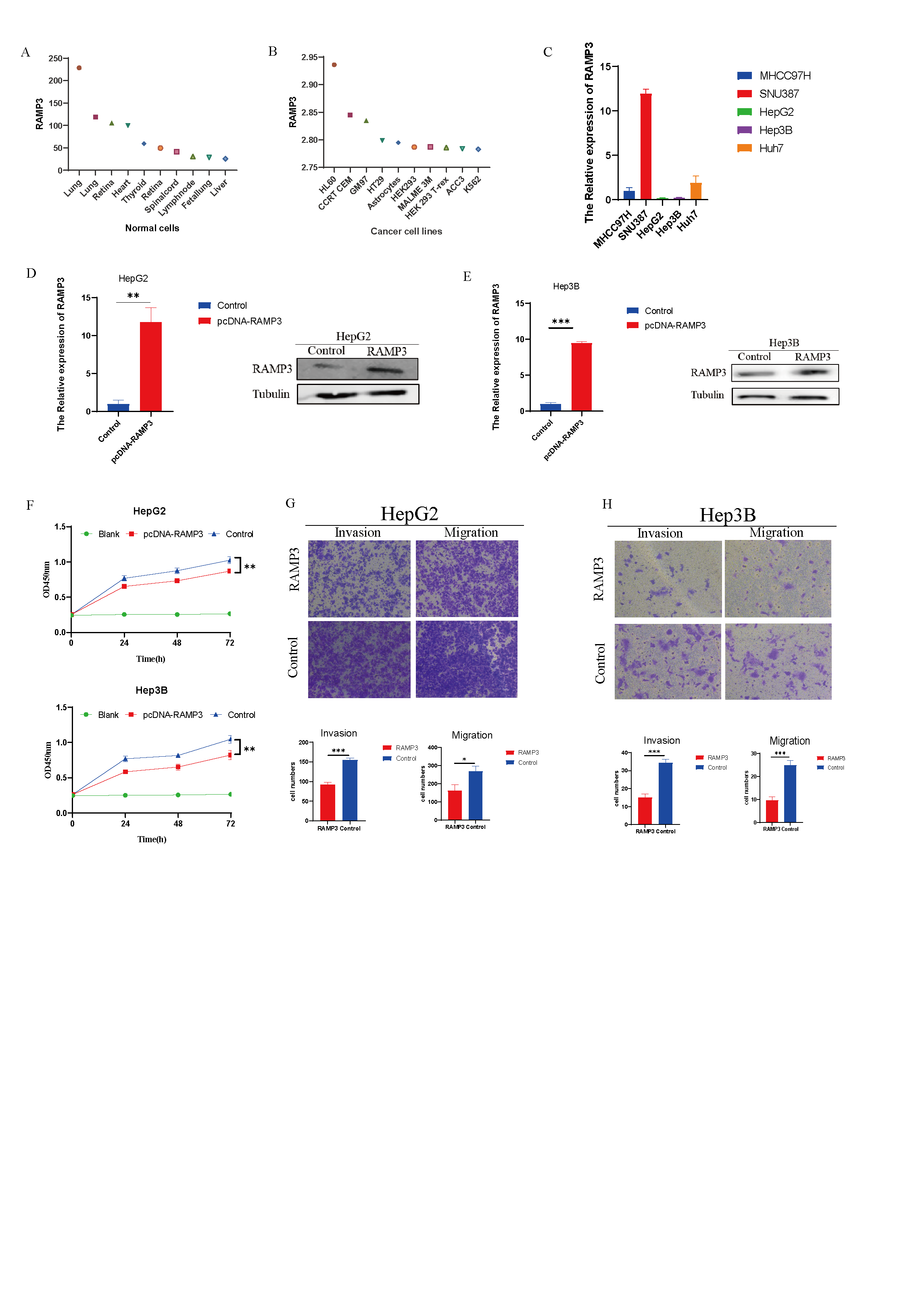

Supplement: Supplementary Table 1 — Genes from TCGA that are differentially expressed in liver cancer tissue and normal tissue. [file DataSheet_1.zip › supplemengtary material/Additional file 5/figS5.tif]

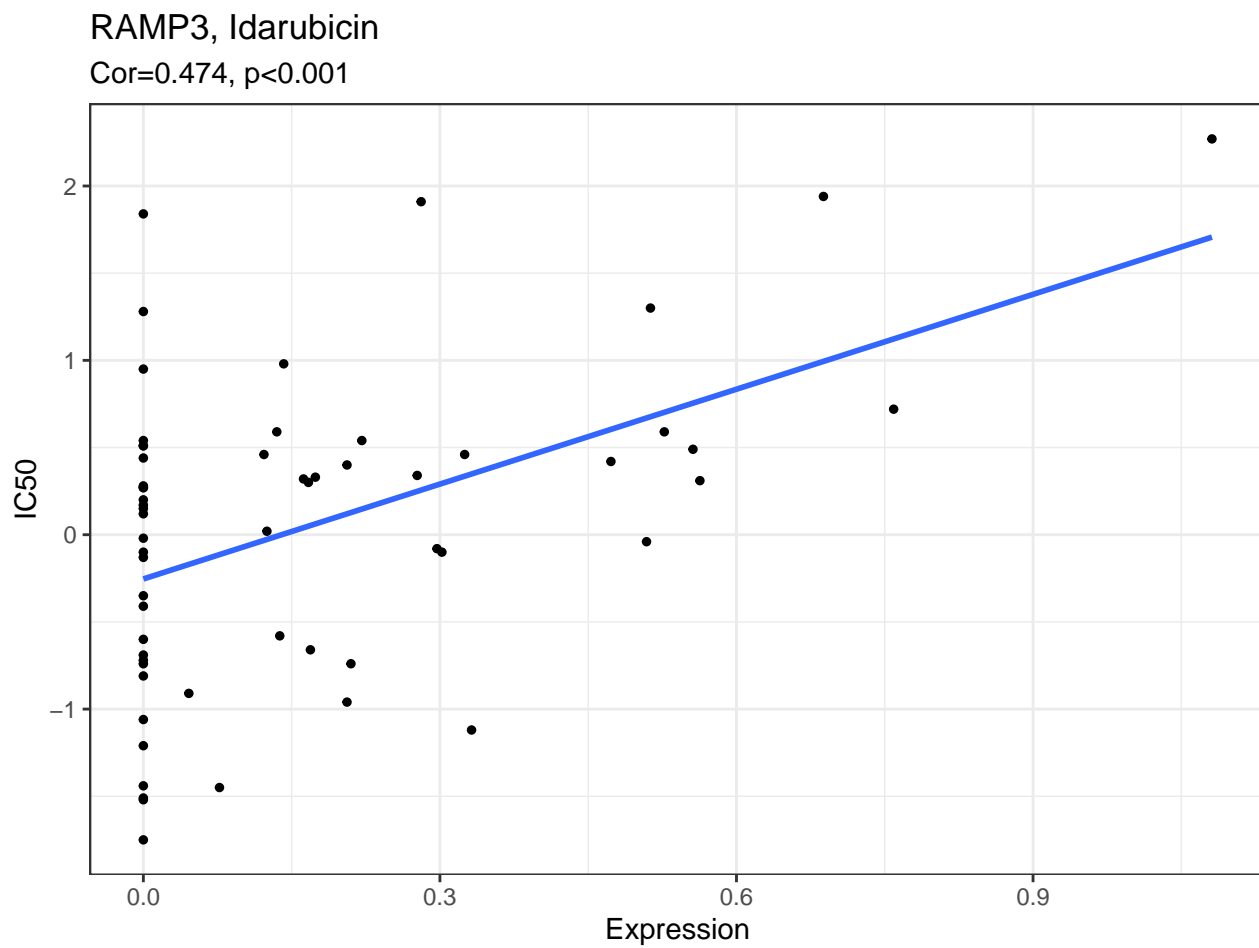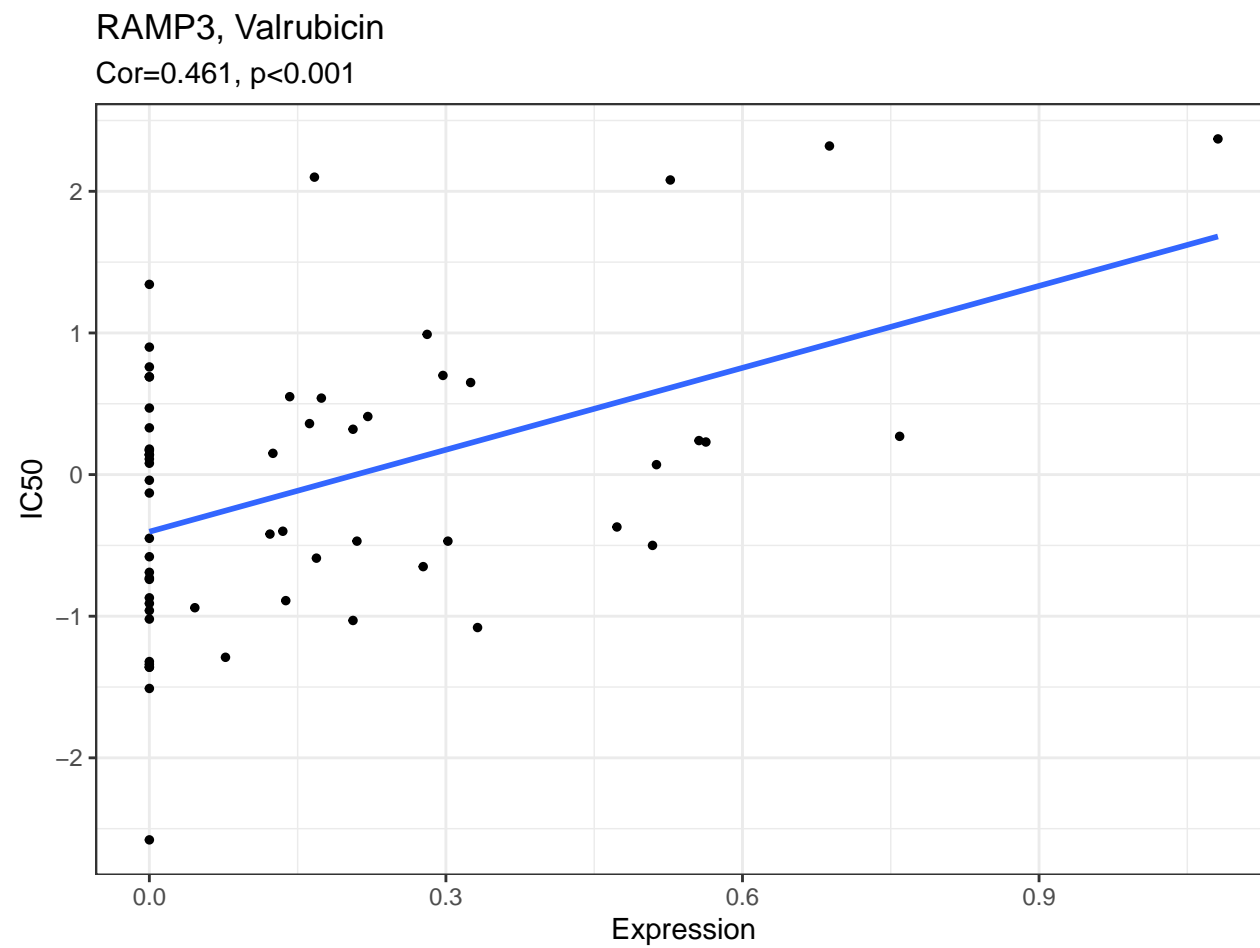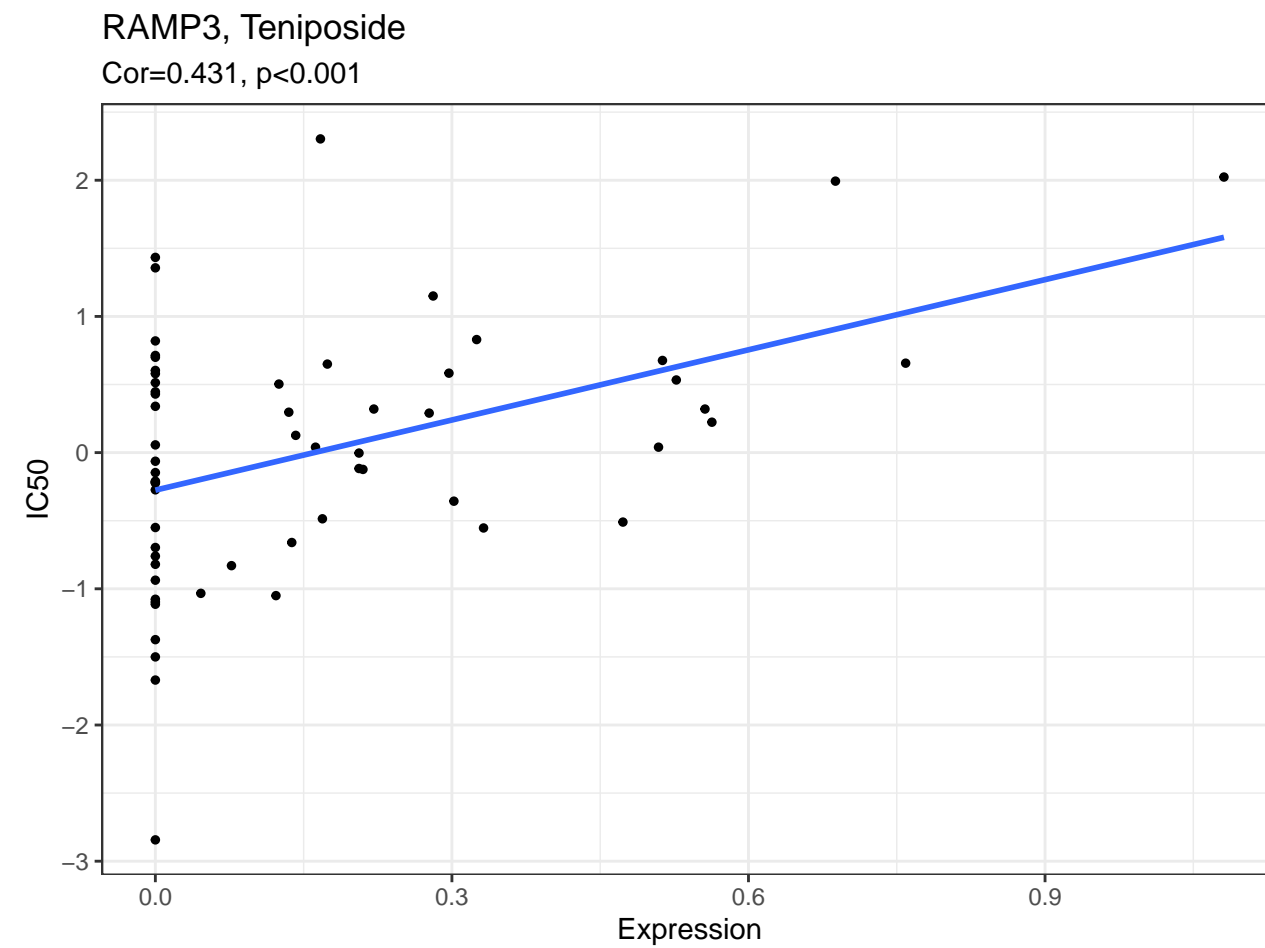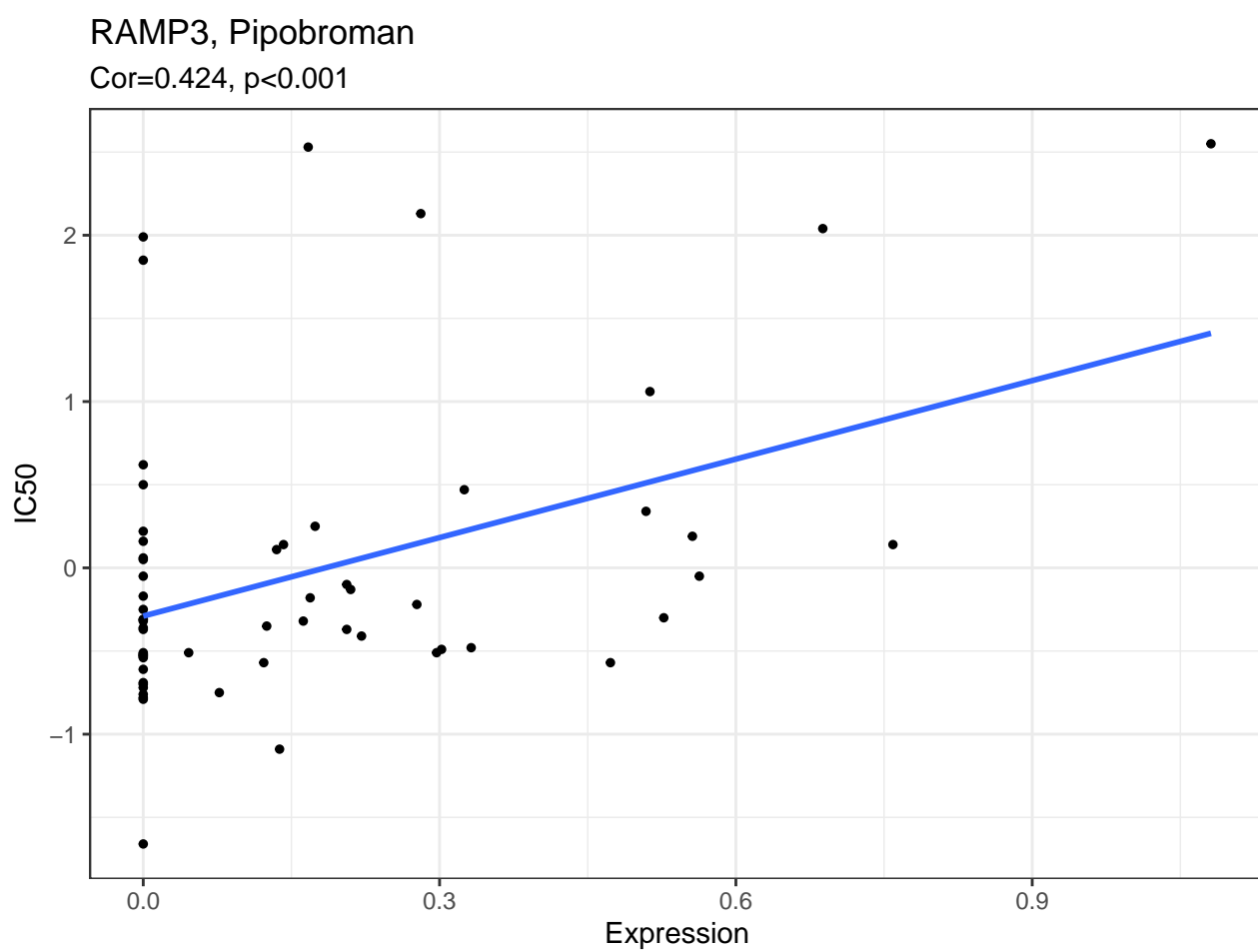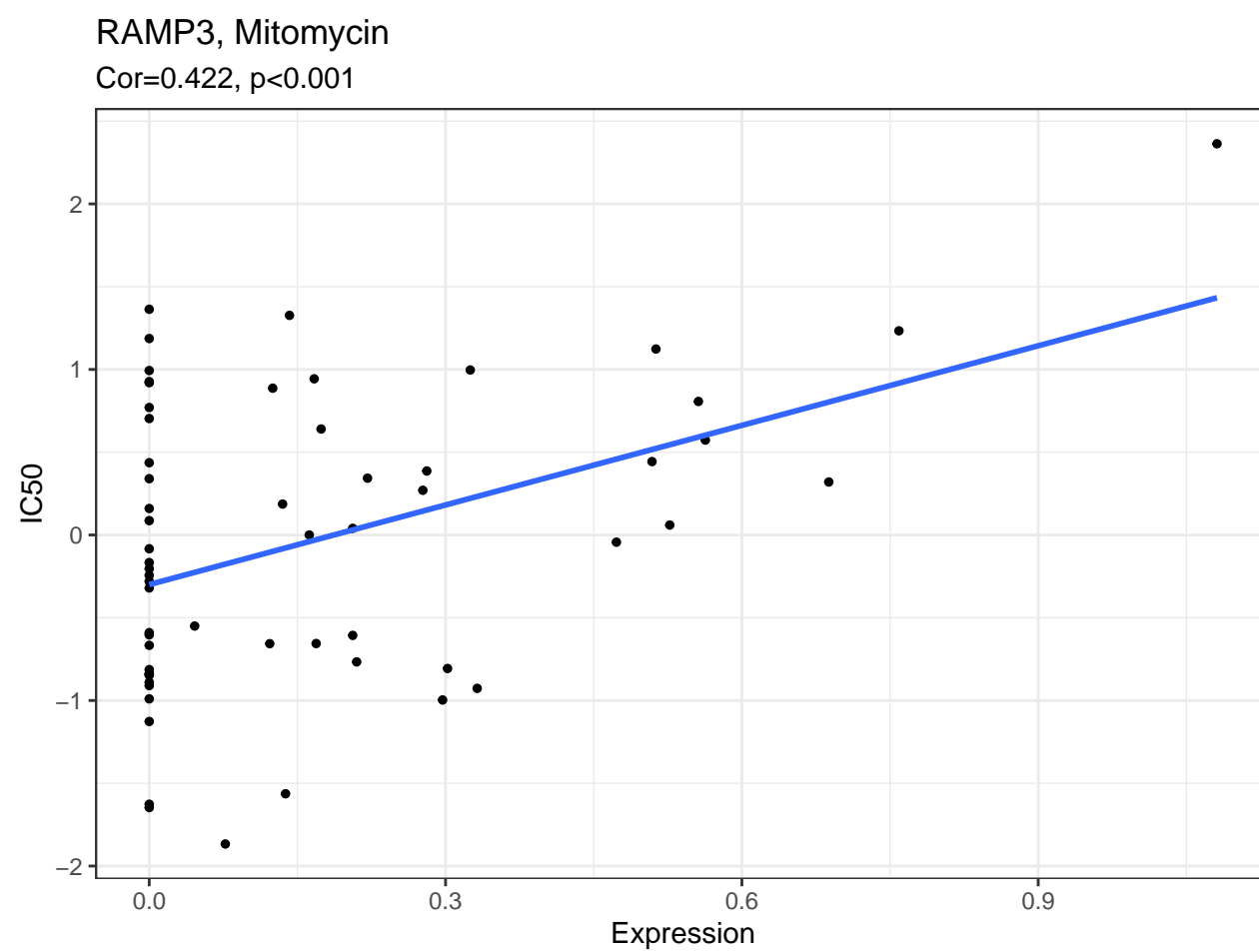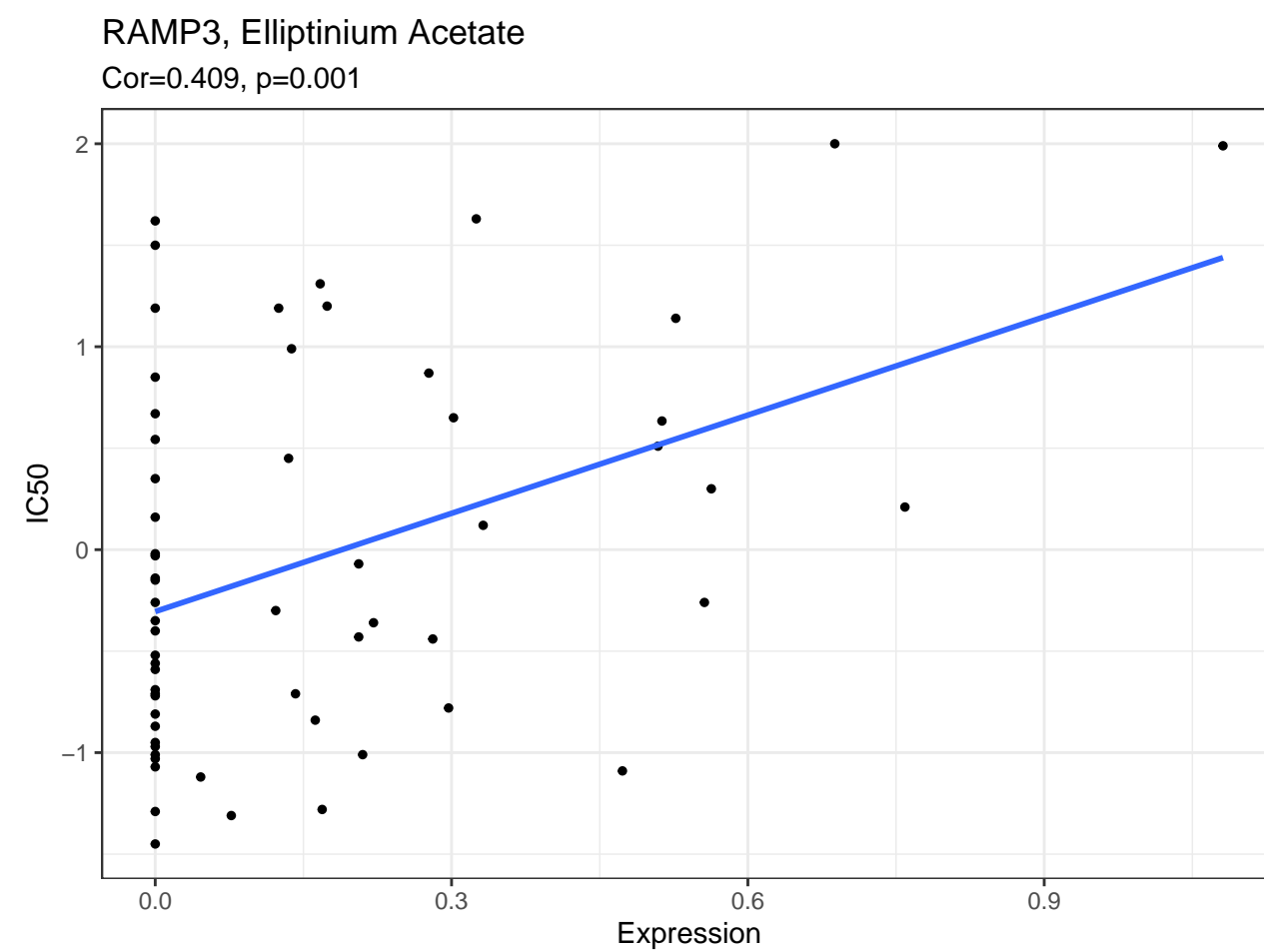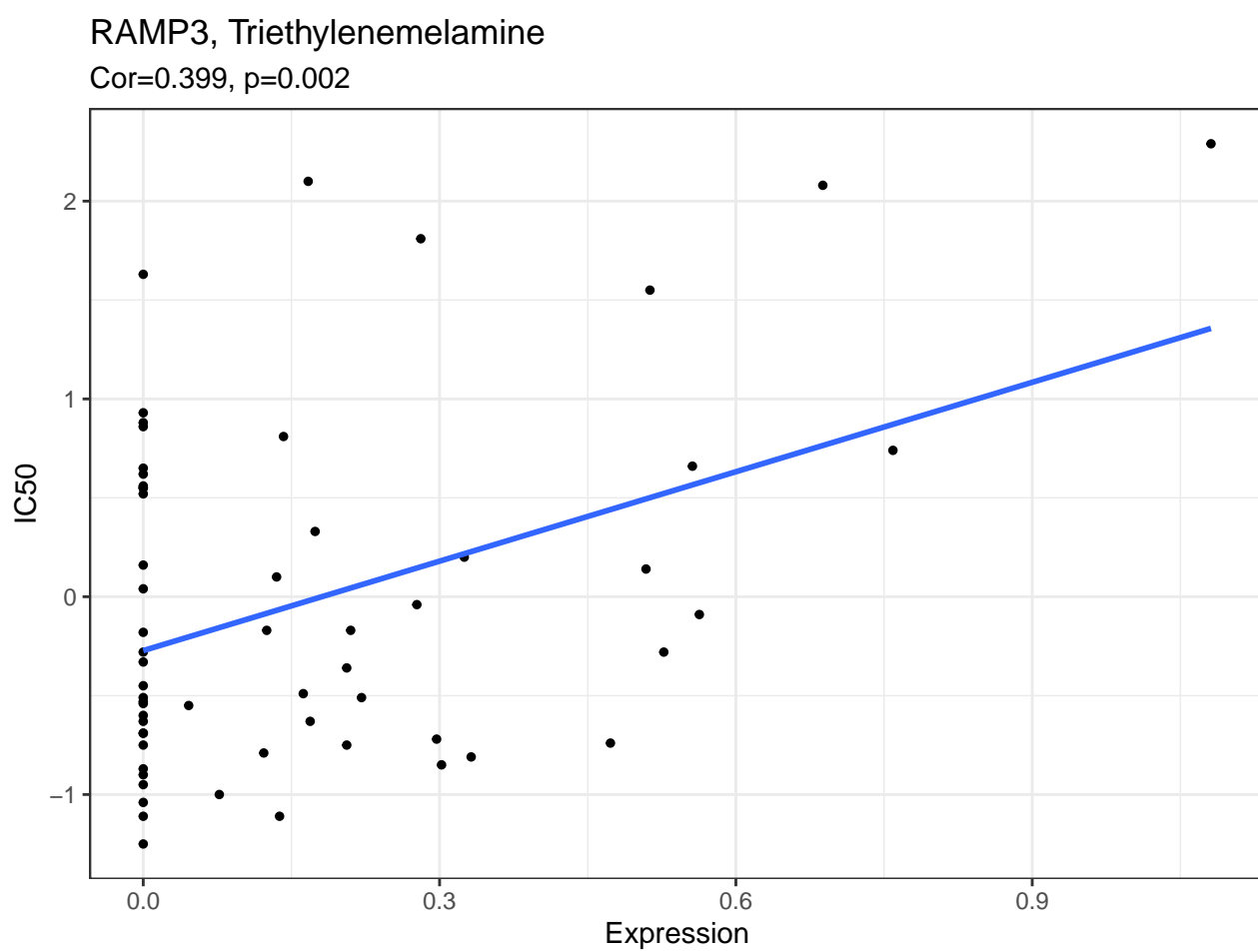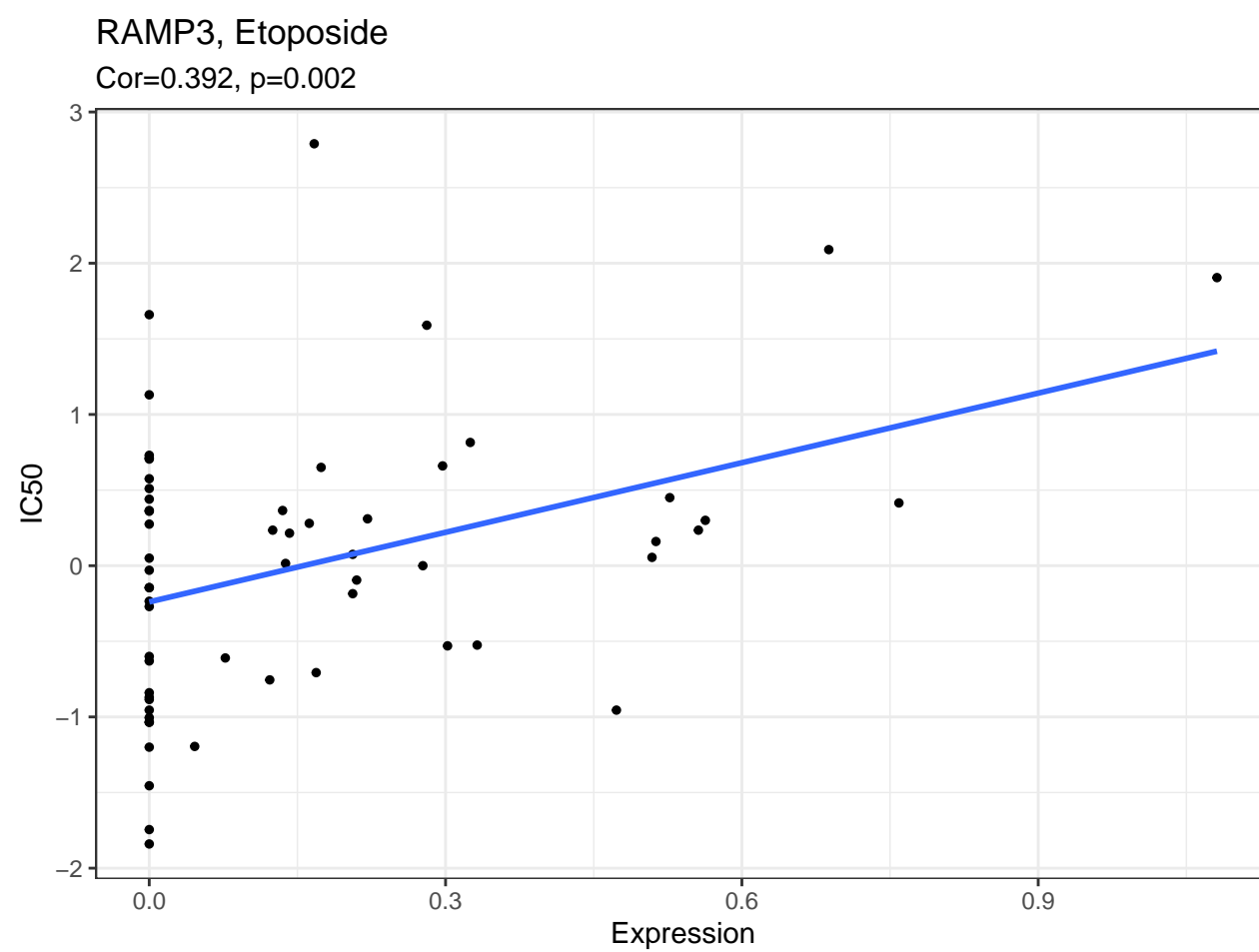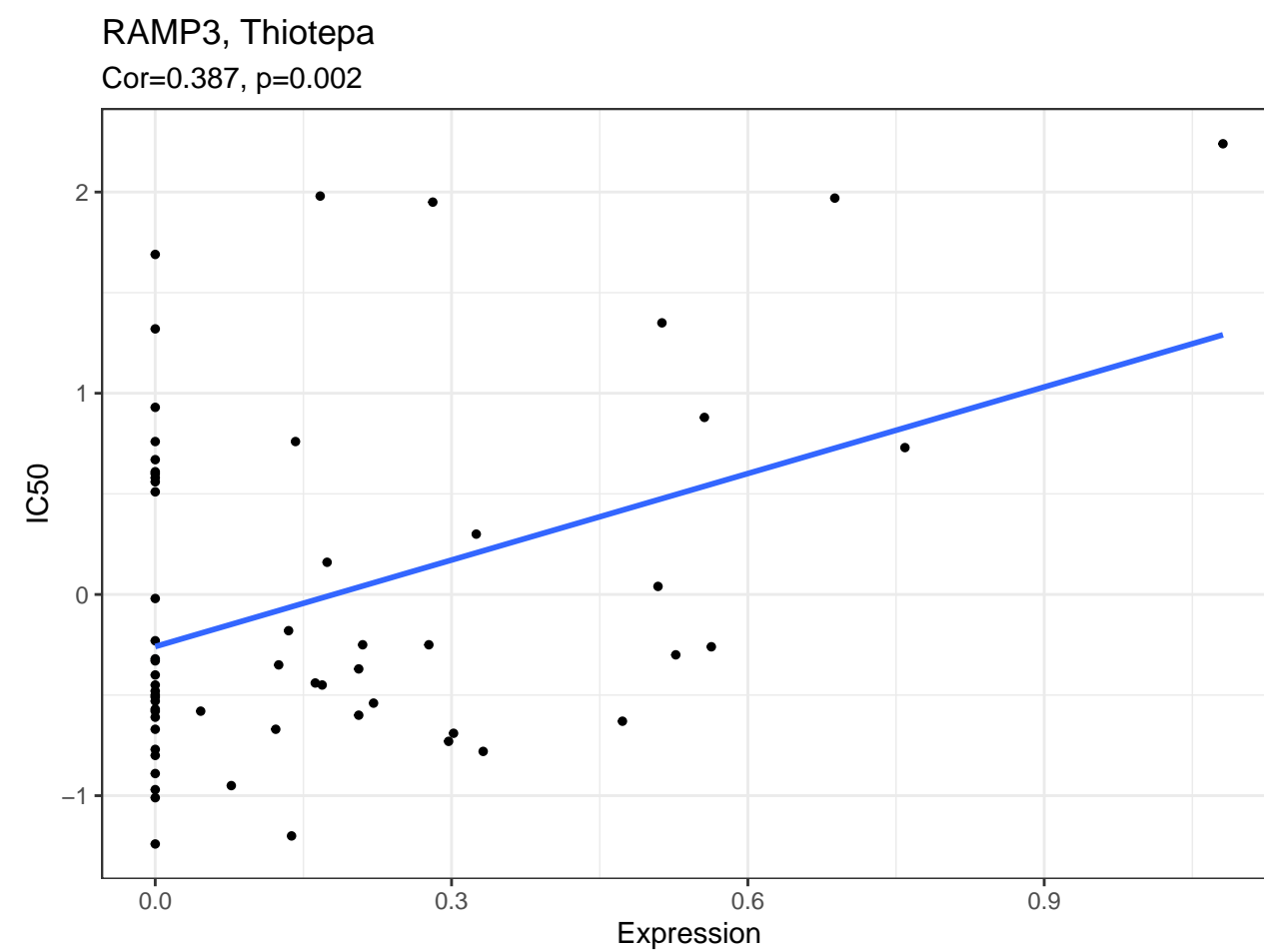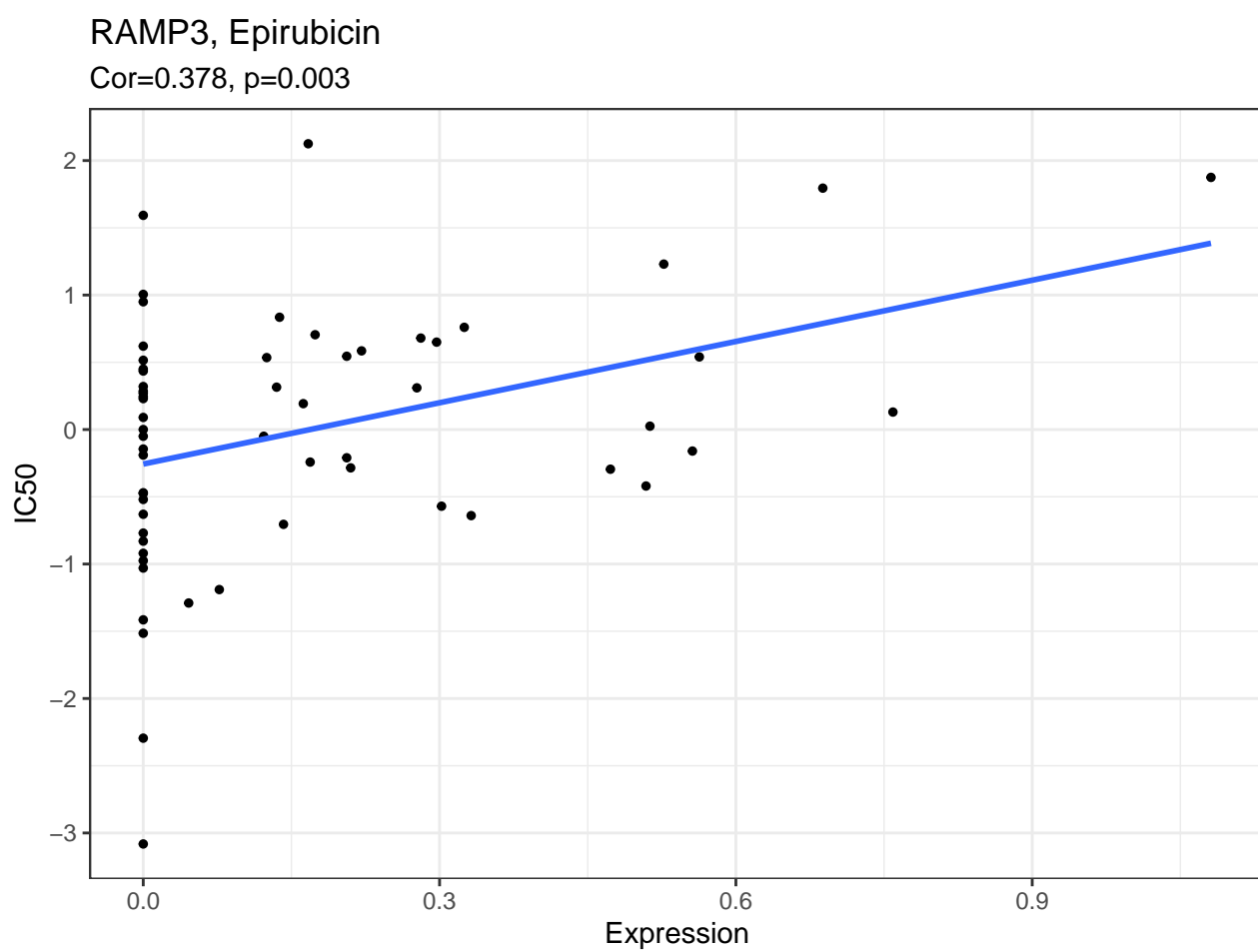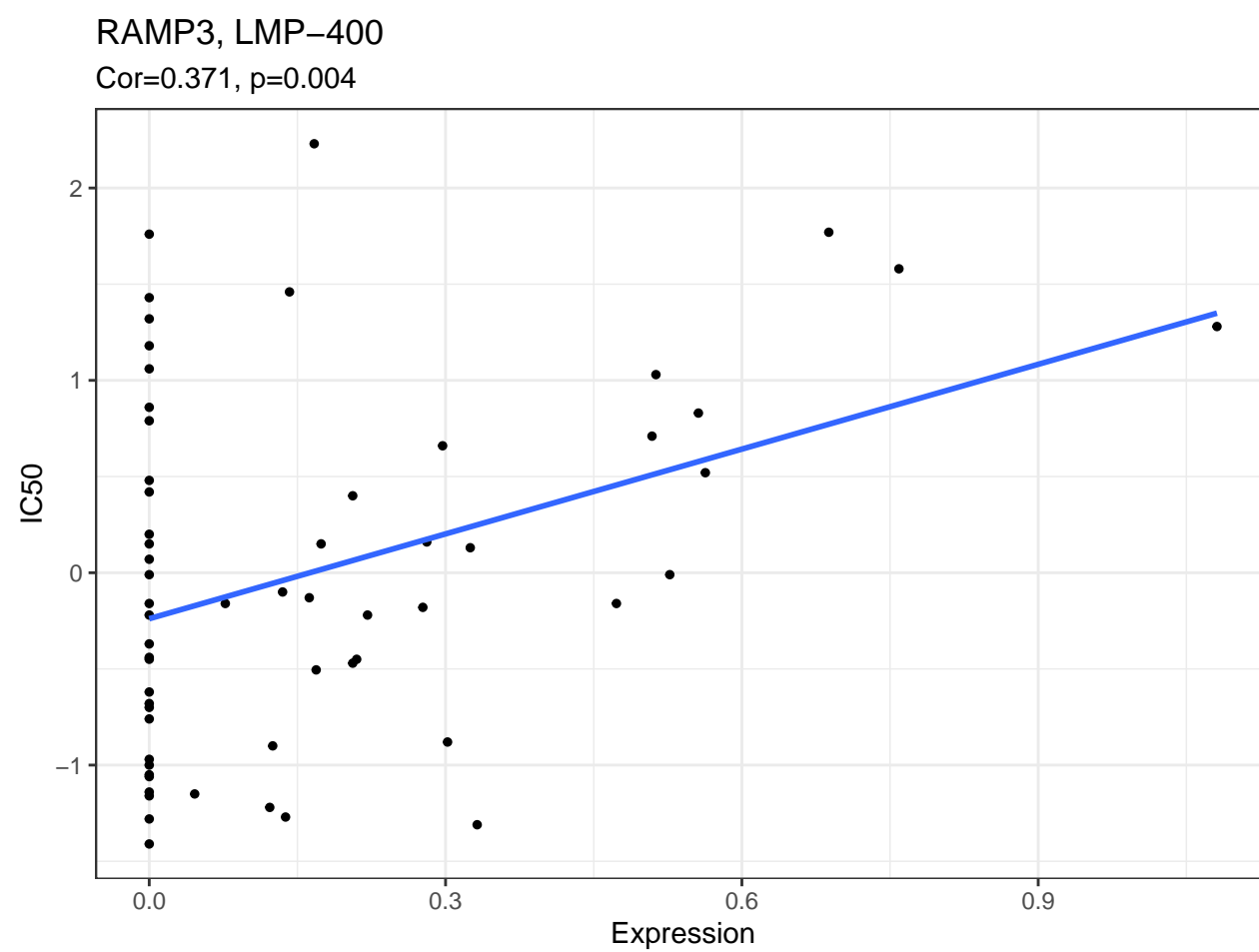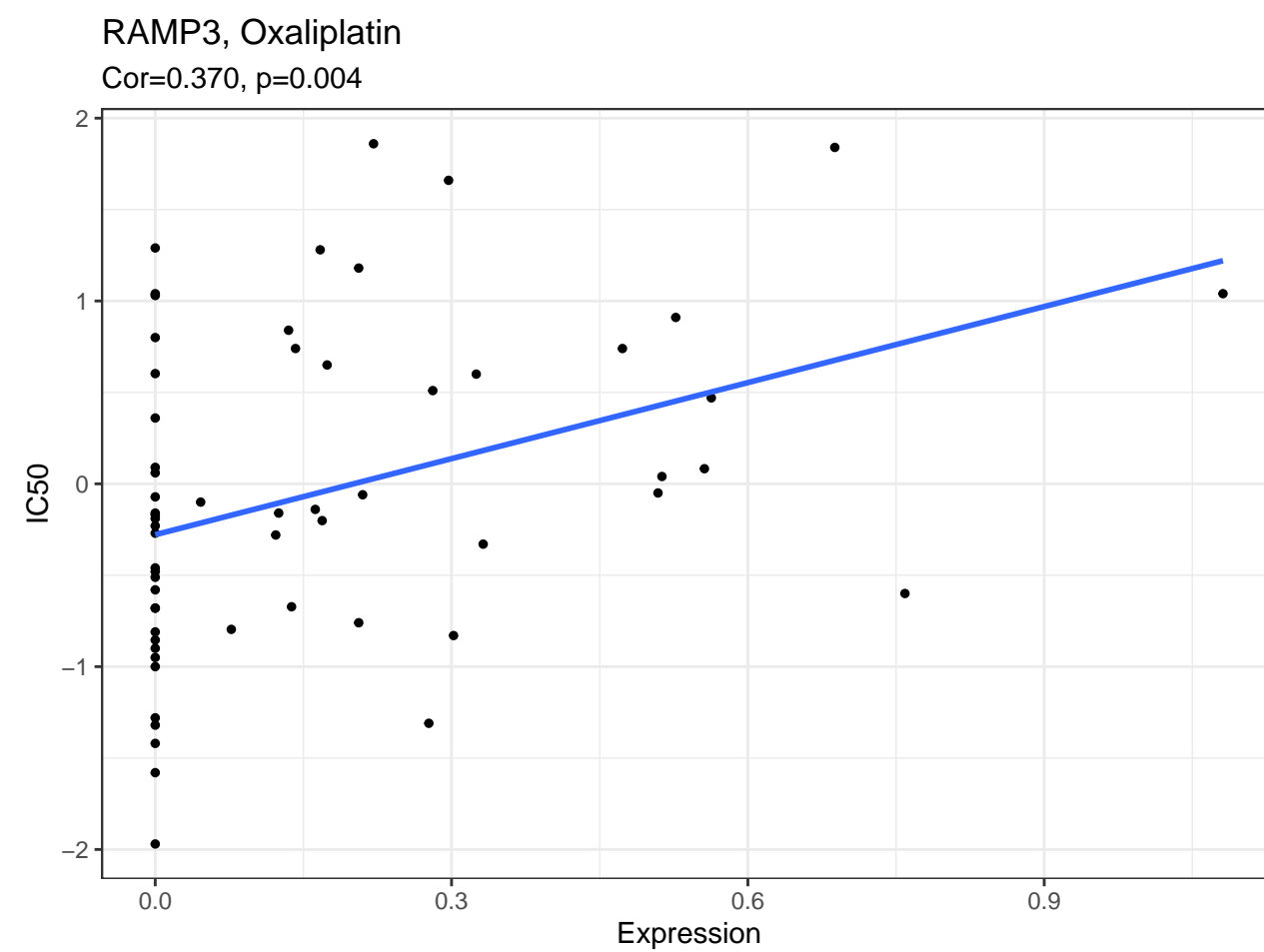

Supplement: Supplementary Table 1 — Genes from TCGA that are differentially expressed in liver cancer tissue and normal tissue. [file DataSheet_1.zip › supplemengtary material/Additional file 6/figs6.pdf]
